# Maturational Stage-Dependent Contributions of the Cav3.2 T-Type Calcium Channel to Dentate Gyrus Granule Cell Excitability

**DOI:** 10.1523/ENEURO.0423-24.2025

**Published:** 2025-04-01

**Authors:** Anne-Sophie Sack, Esperanza Garcia, Terrance P. Snutch

**Affiliations:** ^1^Michael Smith Laboratories, University of British Columbia, Vancouver, British Columbia V6T 1Z4, Canada; ^2^Djavad Mowafaghian Centre for Brain Health, University of British Columbia, Vancouver, British Columbia V6T 1Z3, Canada

**Keywords:** dentate gyrus, excitability, granule cells, T-type channels

## Abstract

T-Type calcium channels shape neuronal excitability driving burst firing, plasticity, and neuronal oscillations that influence circuit activity. The three biophysically distinct T-type channel subtypes (Cav3.1, Cav3.2, Cav3.3) are differentially expressed in the brain, contributing to divergent physiological processes. Cav3.2 channels are highly expressed in the dentate gyrus (DG) of the hippocampus, and mice lacking Cav3.2 [knock-out (KO)] exhibit impairments in hippocampal dependent learning and memory tasks, as well as attenuated development of pilocarpine induced epilepsy. Owing to neurogenesis, granule cells (GCs) are continuously added to the DG, generating a heterogeneous population of maturational stages with distinct excitability. While initial studies identified the role of Cav3.2 in mature GC burst firing, its functional relevance in the intrinsic excitability of different GC subpopulations has not yet been examined. In this study, we used juvenile Cav3.2 KO mice to examine the contributions of Cav3.2 channels to GC excitability at three different stages of maturation. We recorded from cells throughout the GC layer using their electrophysiological and morphological features to allocate GCs into immature, intermediate, and mature groups. In immature GCs, loss of Cav3.2 channels reduced the proportion of cells that fired low-threshold calcium spikes. Conversely, Cav3.2 KO increased excitability in regular spiking intermediate and mature GCs, enabling higher-frequency firing, with little impact on the frequency-dependent response. Overall, this study shows that Cav3.2 channels differentially regulate GC excitability throughout maturation and suggest that calcium influx via Cav3.2 may have maturation-dependent contributions to DG processes such as GC survival, integration, and memory encoding.

## Significance Statement

The generation of new granule cells (GCs) throughout life provides the dentate gyrus (DG) the ability to modify a preexisting circuit. Throughout maturation, GCs undergo substantial changes in morphology and intrinsic excitability prior to becoming indistinguishable to developmentally born GCs. This process occurs over several weeks, during which the unique properties of GCs at specific maturational periods distinctly shape the DG circuit. Here, we provide evidence that Cav3.2 T-type channels contribute to excitability early during GC maturation and shape firing patterns as GCs mature. These results indicate that alterations in Cav3.2 at specific maturational stages may have different consequences on DG excitability, impacting its functions in the hippocampal circuit including those implicated in epilepsy.

## Introduction

Low-threshold-activated T–type calcium channels function as important regulators of neuronal excitability. Opening in response to small membrane depolarizations, T-type channels generate rapidly inactivating currents that contribute to subthreshold signaling (Hutcheon et al., 1994) as well as shaping firing patterns such as high-frequency bursting (Llinás and Jahnsen, 1982; Kim et al., 2001; Vickstrom et al., 2020). In the brain, T-type channels are a critical component of circuit activity contributing to the generation of rhythmic oscillations such as those occurring in the thalamus during sleep (Hughes et al., 2002) and in the inferior olive concerning motor coordination (Choi et al., 2010). Alterations in the expression or kinetics of T-type channels can also drive pathological hyperexcitability and network synchrony thought to occur in disorders like epilepsy (Becker et al., 2008; Powell et al., 2009; Cain and Snutch, 2013).

A signature feature of T-type channel expression in neurons is the generation of low-threshold calcium spikes (LTCS), a slow membrane depolarization initiated from hyperpolarized membrane potentials (Jahnsen and Llinás, 1984). While all three T-type channel subtypes can generate LTCS, key differences in their voltage-dependent properties, kinetics, and somatodendritic expression promote distinct contributions to excitability (Chemin et al., 2002a; McKay et al., 2006; Tscherter et al., 2011; Aguado et al., 2016). In the absence of T-type channel subtype-specific blockers, knock-out (KO) mice have enabled the identification of subtype-specific roles to circuit functions and associated behaviors (Kim et al., 2001; Lee et al., 2004; Becker et al., 2008; Park et al., 2010; Chen et al., 2012; Astori and Luthi, 2013; Gangarossa et al., 2014).

The Cav3.2 T-type channel subtype, encoded by the pore-forming subunit gene *cacna1h*, is expressed throughout the hippocampus with particularly high expression in the dentate gyrus (DG; Talley et al., 1999; McKay et al., 2006; Aguado et al., 2016; Bernal Sierra et al., 2017). Behavioral evidence has implicated the functional role of Cav3.2 channels in the hippocampus as Cav3.2 KO mice exhibit impairments in contextual discrimination tasks and anxiety (Chen et al., 2012; Gangarossa et al., 2014). Furthermore, Cav3.2 KO mice also show reduced pathological hallmarks in the hippocampus and seizure severity in experimental temporal lobe epilepsy (Becker et al., 2008). In the DG of the hippocampus, Cav3.2 channels have been show to underlie burst firing in the principal cells of the DG, mature granule cells (GCs; Dumenieu et al., 2018). However, as a result of the continuous addition of neurons to the DG via neurogenesis, GCs are a heterogeneous population with varying degrees of maturation (Zhao et al., 2006). Immature GCs also shape circuit activity and contribute to processes such as learning and memory (Kee et al., 2007; Gu et al., 2012; Nakashiba et al., 2012; Temprana et al., 2015; Drew et al., 2016) and the aberrant circuit reorganization linked to temporal lobe epilepsy (Overstreet-Wadiche et al., 2006b; Jessberger et al., 2007; Walter et al., 2007; Kron et al., 2010; Althaus et al., 2016). Throughout maturation, GCs undergo extensive morphological and electrophysiological changes, progressing from newborn GCs that fire limited spikes, through a transient period of high intrinsic excitability, before reaching the characteristic low intrinsic excitability of mature GCs (Espósito et al., 2005; Mongiat et al., 2009; Heigele et al., 2016). Given that the density and distribution of ion channels dynamically develop throughout maturation, the contributions of Cav3.2 channels may depend on the maturational stage. In particular, T-type channel–mediated LTCS are present in immature GCs ∼1–3 weeks post mitosis (Ambrogini et al., 2004; Schmidt-Hieber et al., 2004). However, it is unknown whether Cav3.2 specifically impacts GC excitability prior to reaching maturation; therefore, the influence of Cav3.2 channels on DG activity and functions remains unclear.

In the current study, we used Cav3.2 KO mice to determine whether Cav3.2 differentially shapes GCs excitability throughout maturation. In order to examine the contribution of Cav3.2 to the phenotypic features associated with distinct maturational stages, we recorded from cells located throughout the GC layer (GCL) and allocated them into discrete maturational groups (immature, intermediate maturity, and mature) defined by their electrophysiological characteristics, morphology, and expression of proteins. We identified a maturational stage-dependent role of Cav3.2 channels in GC excitability, contributing to the generation of LTCS in immature GCs while shaping the firing frequency in intermediate and mature GCs. Together these results provide evidence for Cav3.2 channels in physiological functions and pathological disorders associated with the DG.

## Materials and Methods

### Animals

All experimental procedures were performed in accordance with the University of British Columbia Animal Care Committee (UBC Protocol A19-0233) following the guidelines provided by the Canadian Council on Animal Care. Male and female C57BL/6J and Cav3.2 KO mice were purchased from Jackson Laboratory (C57BL/6J strain number, 00664 and B6; 129-Cacna1h^tm1Kcam^/J stain number, 013770) and backcrossed for two generations to refresh the colony following Jackson Laboratory guidelines (https://www.jax.org/news-and-insights/jax-blog/2018/april/how-to-refresh-your-mutant-or-transgenic-mouse-strains). Heterozygous mice (Cav3.2^+/−^) were used for breeding to generate Cav3.2 KO (Cav3.2^−/−^) and WT (Cav3.2^+/+^) offspring (Chen et al., 2003), and genotypes were confirmed using the KAPA mouse genotyping kit (Sigma-Aldrich KK5621 and KK7102) with custom primers. Animals were housed in a 12 h day/night cycle at the University of British Columbia Preclinical Discovery Unit and were given food and water *ad libitum*. Mice pups were weaned at Postnatal Day (P)21 and group housed with standard enrichments (nesting material and a hut). All experiments were performed between ZT1 and ZT12.

### Acute brain slice preparation

Young (3–4-week-old) male and female Cav3.2 KO and WT mice were anesthetized to surgical plane with isoflurane [5% in oxygen (v/v)] using a VetEquip inhalation system and killed with a rodent guillotine. Brains were rapidly removed and placed in ice-cold sucrose cutting solution (in mM): 87 NaCl, 25 NaHCO_3_, 25 glucose, 75 sucrose, 2.5 KCl, 1.25 NaH_2_PO_4_, 0.5 CaCl_2_, and 7 MgCl_2_ bubbled with 95% O_2_:5% CO_2_ gas mixture. The dorsal part of the brain was trimmed at an angle close to 0°, cutting away ∼10–20% of the total brain volume using the “magic-cut” protocol (Bischofberger et al., 2006). The cut side of the brain was glued onto the vibratome stage (VT12000; Leica), and hippocampal sections (300–350 µm thick) were cut from lateral to medial. Slices recovered in sucrose solution at 35°C for 30 min and were subsequently stored at room temperature (21–24°C) until use. Experiments were performed at 22 ± 2°C.

### Whole-cell patch–clamp recordings

Slices were transferred to a recording chamber (RC 26G chamber and 64-0255 Slice Anchor; Warner Instruments) and perfused (flow rate 2 ml/min) for at least 10 mins with artificial cerebral spinal fluid (in mM): 125 NaCl, 25 NaHCO_3_, 25 glucose, 2.5 KCl, 1.25 NaH_2_PO_4_, 2 CaCl_2_, and 1 MgCl_2_ bubbled with 95% O_2_:5% CO_2_ gas at room temperature. DG GCs were visualized with an infrared differential interference contrast with a 40× water immersion objective in a Zeiss Axioscop 2 FSPlus microscope. Patch pipettes (resistance 3–6 MΩ) were pulled from borosilicate capillary glass (Sutter BF150-86-10) with micropipette puller (Sutter Instruments, P-1000). Whole-cell patch–clamp recordings were made from GCs in current clamp with pipettes filled with internal solution (in mM): 130 K-gluconate, 20 KCl, 10 HEPES, 2 MgCl_2_, 4 K_2_ATP, 0.3 NaGTP, and 10 Na_2_-phosphocreatine, pH 7.2 with KOH, (Schmidt-Hieber et al., 2004). Recordings were made from GCs located throughout the GCL and were subsequently grouped into maturational stages using previously identified electrophysiological characteristics described in the results (Schmidt-Salzmann et al., 2014). Recordings were performed with Multiclamp 700B amplifier, digitized using the Axon Digidata 1550B acquisition system, and pClamp Software (version 11, Molecular Devices). Current-clamp recordings were low-pass filtered at 10 kHz and digitized at 50 kHz. Series resistance and pipette capacitance were compensated with bridge balance and capacitance neutralization. Cells were discarded if they had an access resistance >35 MΩ or if access resistance or bridge balance changed >15% during recording. Resting membrane potential (RMP) was corrected with small current injections when required. Data not corrected for liquid junction potential (experimentally measured using a 3 M KCl 3% agar bridge as 9.7 mV).

### Immunostaining

In a subset of cells, the recording pipette was filled with the tracer Neurobiotin-488 (0.2% w/v in the internal solution; (VectorLabs catalog #SP-1125-2) to classify neurons based on morphology and expression of maturation markers. After electrophysiological recordings, neurons were loaded with Neurobiotin-488 by applying hyperpolarizing current pulses for ∼20 mins before the electrode was carefully withdrawn. Slices were fixed in 4% paraformaldehyde in 0.1 M phosphate-buffered saline (PBS; in mM): 137 NaCl, 2.7 KCl, 10 Na_2_HPO_4_, and 1.8 KH_2_PO_4_, pH 7.4, for 2–4 h at 4°C. Immunostaining protocol was adapted from Swietek et al. (2016). Free-floating sections were washed with PBS and then incubated with 0.3% Triton X-100 and 3% donkey serum for 30 min at room temperature. Sections were then stained with sheep anti-DCX (8 µg/ml; Novus Biologicals catalog #AF10025; 1% donkey serum in PBS) for three nights at 4°C. Following a wash in PBS, slices were incubated overnight in the secondary donkey anti-sheep Alexa Fluor 633 (1:400; Invitrogen catalog #A21100) at 4°C. Finally, slices were incubated with conjugated primary antibody mouse anti-NeuN Alexa Fluor 555 (1:100; Merck Millipore catalog #MAB377A5) overnight at 4°C. Sections were mounted onto coverslips in VECTASHIELD (VectorLabs). Immunofluorescence was imaged using the Olympus FV1000 Laser Scanning/two-photon confocal microscope at the University of British Columbia Bioimaging facility (RRID: SCR_021304). Background fluorescence due to intracellular dye leakage upon pipette removal was corrected from final images for clarity when required.

In an attempt to localize Cav3.2 channels in DG GC subpopulations, three Cav3.2 channel antibodies with distinct epitopes were tested in DG slices. In subset of slices, an antigen retrieval method was also tested (Cheng et al., 2019). The experimental approach and immunostaining results are shown in Extended Data Figures 1-1 and 1-2. In general, Cav3.2 immunolabeling was not specific enough to unequivocally demonstrate the localization of the channel protein, with negative staining depending on the antibody. Notably, the Cav3.2 monoclonal antibody (Santa Cruz Biotechnology: SC-25691) used by other labs including a study that showed staining of Cav3.2 channels in the DG (Yabuki et al., 2021) has now been discontinued, and so we were unable to test it.

**Figure 1.**
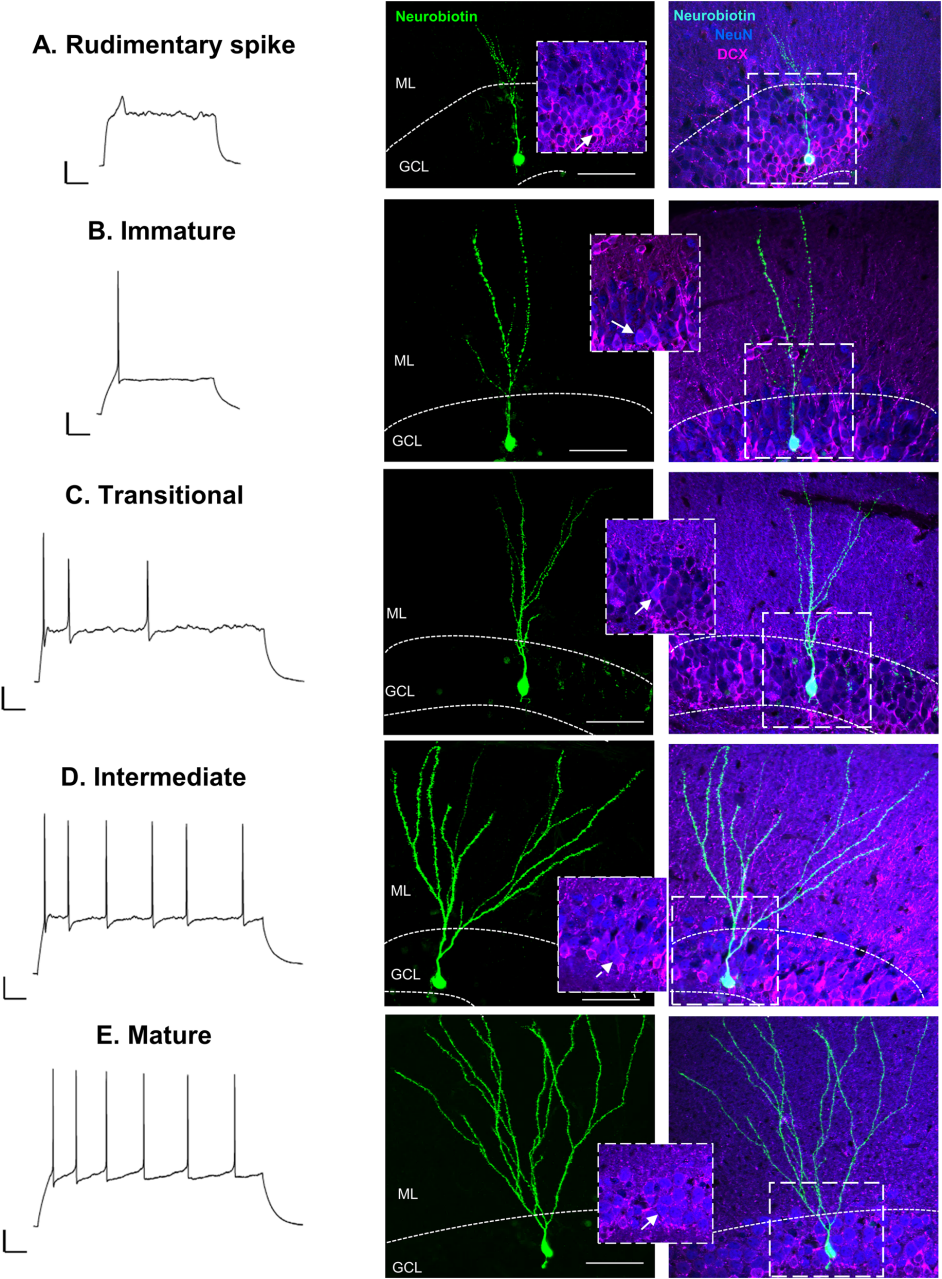
Morphological and electrophysiological maturation of DG GCs. The morphological and functional heterogeneity of GCs is illustrated by the different action potential firing patterns correlated with distinct morphological complexity, as revealed by Neurobiotin-488 staining. Representative responses to a suprathreshold depolarizing current stimulus (left traces) and the corresponding confocal images of Neurobiotin-488 labeling (green, middle panels) used to delineate the morphology of recorded GCs are shown to illustrate different levels of dendritic arbor complexity, correlated with distinct firing pattern. Overlay images (right panels) of neurobiotin (cyan) with DCX (pink) and neuronal nuclear protein (NeuN, blue) show that immunolabeling for DCX and NeuN was positive in the rudimentary spiking cell (***A***), immature (***B***), and transitional cell (***C***; see inserts), which also show limited dendritic arborization. Intermediate (***D***) and mature (***E***) GCs displayed a mature morphology with extensive arborization that reached the molecular layer and were positive for NeuN. Weak staining for DCX was detected in the intermediate GC but nondetectable in the mature GC. Inserts show merged images of DCX and NeuN indicating colocalization in the recorded cell (arrow). Dotted line indicates border of granule cell layer (GCL). [Molecular layer (ML); scale bar, 50 µm]. Immunohistochemistry protocols used to localize Cav3.2 channels in the DG with representative images are shown in Extended Data Figures 1-1 and 1-2.

10.1523/ENEURO.0423-24.2025.f1-1 Figure 1-1 **Flowchart of immunohistochemistry protocols used to localize Cav3.2 channels in the DG.** Following transcardial perfusion with paraformaldehyde (PFA), brains were dissected, placed in 4% PFA overnight at 4 °C and then sucrose (10% overnight, followed by 30% overnight) for cryoprotection. Fixed brains were subsequently embedded in optimal cutting temperature (O.C.T) compound and stored at -80 °C. Tissue was sliced into 40  µm thick coronal sections or magic cut sections using a cryostat. Sections were washed in PBS and stored in storage solution (0.81 M sucrose, 30% ethylene glycol in 0.1 M PBS) at -80 °C until use (Potts et al., 2020). Free floating sections were washed with PBS prior to either antigen retrieval or directly to permeabilization and blocking (RT: room temperature). Download Figure 1-1, TIF file.

**Figure 2.**
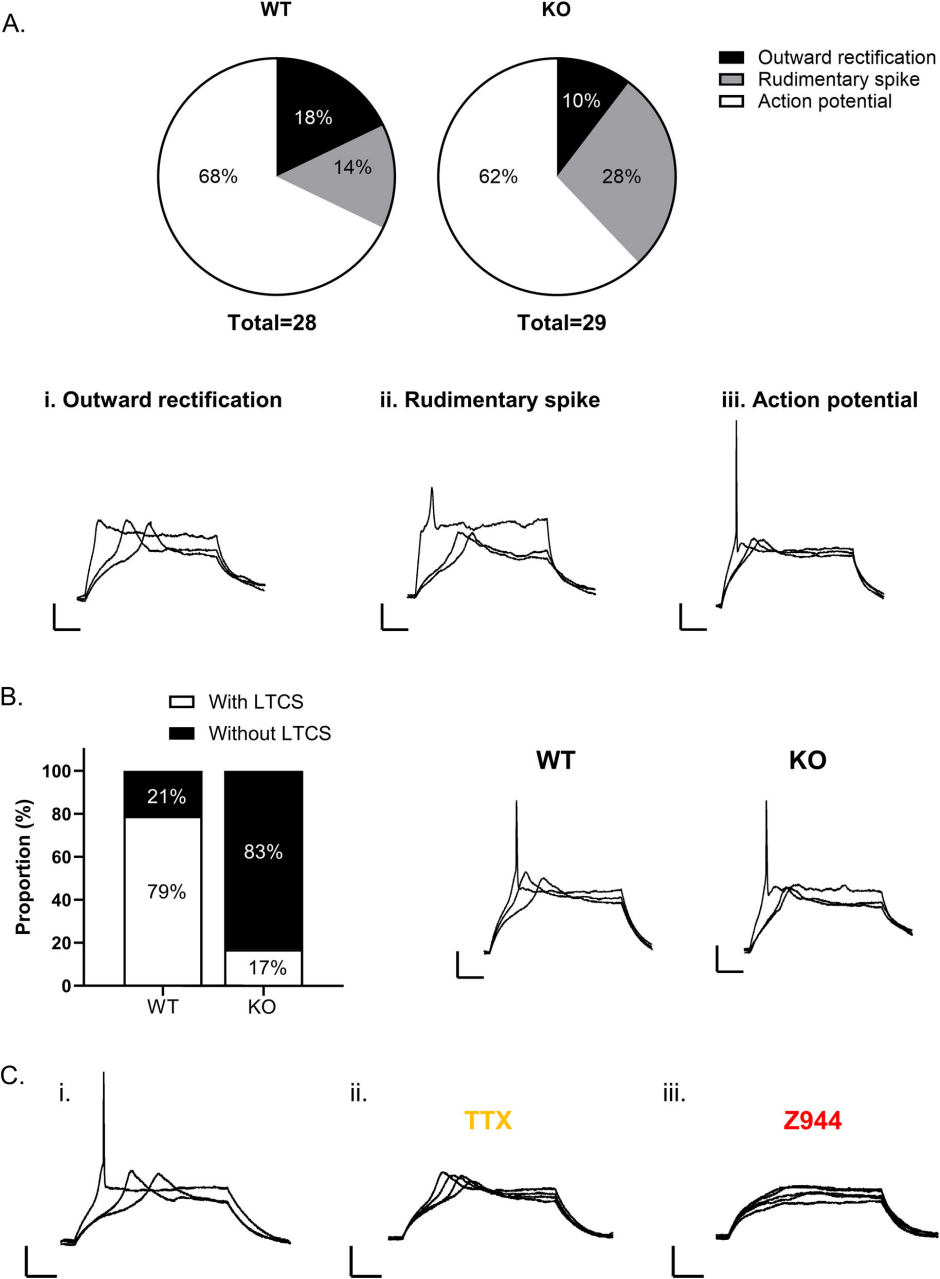
T-Type channel-mediated LTCS in immature GCs. Responses evoked by current stimulation were used to characterize the intrinsic membrane properties of cells in the inner GCL in order to compare populations from Cav3.2 KO, relative to WT mice. Cells were grouped in three main categories (***A***): cells displaying outward rectification (***i***), cells exhibiting a rudimentary spike (***ii***), and cells generating one or a few action potentials in response to small depolarizing current steps (***iii***). In both WT and Cav3.2 KO, predominant subtypes were cells that fired few action potentials (***A***). Example traces show LTCS observed in a subpopulation of all three cell types. LTCS were most frequently observed in immature GCs, preceding action potential firing (traces panel ***B***). The proportion of GCs with LTCS in Cav3.2 KO mice was smaller than in WT (Fisher's exact test *p* = 0.0002; bar graph, ***B***). Representative recordings from WT slices show the response of an immature neuron (***Ci***) to current steps of increasing intensity, characterized by a slow LTCS that initiates with a shorter latency as the stimulus intensity increases, and a fast sodium-dependent action potential that is suppressed by the application of 1 µM TTX (***Cii***). LTCS was insensitive to TTX (***C****ii*) but completely blocked by pan-T–type channel blocker Z944 (1 µM) (***Ciii***).

10.1523/ENEURO.0423-24.2025.f1-2 Figure 1-2 **Immunohistochemical staining in the DG, comparing different antibodies targeting Cav3.2 channels.** Three different commercial antibodies recognizing distinct epitopes were systematically tested in both WT and KO mice brain slices. The Neuromab antibody (A) did not show any immunoreactivity for Cav3.2 channels in the DG or other brain regions examined (not shown). Antigen retrieval (AR) method (B) resulted in non-specific background fluorescence with no improvement of Cav3.2 immunolabeling with Neuromab antibody. Although immunofluorescence was observed in GCs using the Santa Cruz antibody (C), the staining matched and overlapped entirely with that obtained using doublecortin (DCX), indicating cross-reactivity (insert shows superimposed Cav3.2 DCX staining). Lastly, by using the Alomone antibody (D), non-specific immunolabeling was detected in hilar cells and some inner GCs. All antibodies showed a similar pattern in slices from KO mice (not shown). We further observed diffuse, non-specific staining in other regions of the brain known to express Cav3.2 channels (not shown) (ML = molecular layer. GCL = granule cell layer). Download Figure 1-2, TIF file.

### Data analysis

GCs were categorized based on input resistance (immature >1 GΩ, intermediate >400 MΩ, mature <400 MΩ; Schmidt-Salzmann et al., 2014). Current–voltage relationships were measured using the following protocols: immature, 500 ms steps from −5 to 30 pA Δ2 pA, and intermediate and mature, 1,000 ms steps from −40 to 150 pA Δ10 pA. Input resistance was calculated from the linear part of the *I*–*V* near RMP. The action potential rate of rise was measured from the first action potential at rheobase. Interspike interval (ISI) was used to classify burst spiking or regular spiking cells at rheobase +60 pA, a stimulation intensity that clearly distinguished GC burst spiking from regular spiking (burst spiking GCs first ISI <30 ms, corresponding to frequencies ≥30 Hz). This stimulation intensity was used for all steady-state relationships measured. Suprathreshold frequency responses were studied by injecting square pulse currents at a frequency of 4 or 8 Hz at an intensity of 1.5× rheobase Subthreshold frequency-dependent responses were examined using a CHIRP stimulation: 20 pA stimulation and 0–15 Hz in 15 s. The impedance amplitude profile (ZAP) was generated from the ratio of the fast Fourier transform of the voltage response to the fast Fourier transform CHIRP stimulus. Resonance strength was calculated as the ratio of the maximum impedance amplitude to the impedance amplitude at 0.5 Hz, and resonance frequency was measured as the frequency of maximum impedance (Mishra and Narayanan, 2020). Analysis of electrophysiological data was performed with Clampfit 11.2 (Molecular Devices). Statistical analysis and visualization were performed with GraphPad Prism version 10 (GraphPad Software) and Origin 2020 (OriginLab). Comparisons of categorical variables between genotypes were made with Fisher's test. Estimation statistics was used to analyze the data to report the effect size and confidence intervals (CI) along with mean values ± SEM when two groups were compared (Gardner and Altman, 1986; Bernard, 2019; Calin-Jageman and Cumming, 2019; Ho et al., 2019). The left axis of estimation plots shows individual data points, and the right axis shows the difference between means with 95% CI. Statistical comparisons between two groups were made using unpaired Student's *t* test or Mann–Whitney test if normality assumption was not met. Normality assumption was tested using D’Agostino and Pearson’s test. Summary of statistical values comparing two groups are shown in Tables 1–3. Analysis of ISI versus ISI number and number of spikes versus current were assessed with repeated-measure two–way ANOVA or mixed effects analysis if data contained missing values with Geisser–Greenhouse correction. Multiple comparisons were made with Sidak's test. No significant sex differences were observed in excitability parameters measured; thus data from male and female mice were pooled for analysis.

**Table 1.** Comparison of electrophysiological parameters at different maturational stages in WT and Cav3.2 KO GCs

Parameter	Intermediate (mean ± SEM)	Mature (mean ± SEM)	Number of cells	Statistical test	*p* value
WT input resistance (MΩ)	627.1 ± 41.40	290.00 ± 15.81	Intermediate, *n* = 19; mature, *n* = 18	Unpaired *t* test	*p* < 0.0001
KO input resistance (MΩ)	748.10 ± 90.40	283.50 ± 13.19	Intermediate, *n* = 14; mature, *n* = 18	Mann–Whitney test	*p* < 0.0001
WT rheobase (pA)	39.68 ± 2.68	61.50 ± 4.88	Intermediate, *n* = 31; mature, *n* = 20	Mann–Whitney test	*p* < 0.0001
KO rheobase (pA)	40.53 ± 3.46	67.22 ± 3.60	Intermediate, *n* = 19; mature, *n* = 18	Unpaired *t* test	*p* < 0.0001
WT maximum impedance magnitude (MΩ)	1,068 ± 97.22	515.9 ± 39.19	Intermediate, *n* = 9; mature, *n* = 10	Unpaired *t* test	*p* < 0.0001
KO maximum impedance magnitude (MΩ)	1,244 ± 153.4	556.6 ± 88.17	Intermediate, *n* = 5; mature, *n* = 6	Unpaired *t* test	*p* = 0.0028

Summary of statistical analysis.

**Table 2.** Comparison of electrophysiological parameters in WT versus Cav3.2 KO intermediate GCs

Parameter	WT (mean ± SEM)	KO (mean ± SEM)	Number of cells	Statistical test	*p* value
Initial firing frequency in burst spiking GCs (Hz)	49.81 ± 2.50	53.43 ± 4.91	WT, *n* = 12; KO, *n* = 5	Unpaired *t* test	*p* = 0.4775
Average firing frequency (1st ISI to 4th ISI) in regular spiking GCs (Hz)	11.57 ± 0.95	14.67 ± 1.24 Hz	WT, *n* = 13; KO, *n* = 8	Unpaired *t* test	*p* = 0.0614
Average firing frequency in regular spiking GCs (Hz)	9.64 ± 0.56	11.18 ± 0.62	WT, *n* = 13; KO, *n* = 8	Unpaired *t* test	*p* = 0.0925
RMP (mV)	−82.81 ± 0.83	−84.70 ± 0.88	WT, *n* = 25; KO, *n* = 13	Unpaired *t* test	*p* = 0.1607
Input resistance (MΩ)	619.80 ± 45.83	736.70 ± 135.1	WT, *n* = 17; KO, *n* = 8	Mann–Whitney test	*p* = 0.7975
4 Hz cumulative frequency (Hz)	3.85 ± 0.39	4.58 ± 0.25	WT, *n* = 6; KO, *n* = 8	Unpaired *t* test	*p* = 0.1223
4 Hz number of spikes	3.67 ± 0.33	4.25 ± 0.25	WT, *n* = 6; KO, *n* = 8	Unpaired *t* test	*p* = 0.1779
8 Hz cumulative frequency (Hz)	5.02 ± 0.87	5.48 ± 0.29	WT, *n* = 6; KO, *n* = 7	Unpaired *t* test	*p* = 0.6063
8 Hz number of spikes	5.00 ± 0.73	4.43 ± 0.57	WT, *n* = 6; KO, *n* = 7	Unpaired *t* test	*p* = 0.5448
Resonance frequency (Hz)	0.72 ± 0.083	0.653 ± 0.104	WT, *n* = 9; KO, *n* = 5	Mann–Whitney test	*p* = 0.2118
Resonance strength	1.24 ± 0.060	1.06 ± 0.050	WT, *n* = 9; KO, *n* = 5	Unpaired *t* test	*p* = 0.0686
Maximum impedance magnitude (MΩ)	1,068 ± 97.22	1,244 ± 153.4	WT, *n* = 9; KO, *n* = 5	Unpaired *t* test	*p* = 0.3275

Summary of statistical analysis.

**Table 3.** Comparison of electrophysiological parameters in WT versus Cav3.2 KO mature GCs

Parameter	WT (mean ± SEM)	KO (mean ± SEM)	Number of cells	Statistical test	*p* value
Average firing frequency in regular spiking GCs (Hz)	9.36 ± 0.63	11.32 ± 0.58	WT, *n* = 17; KO, *n* = 14	Mann–Whitney test	*p* = 0.0131
RMP (mV)	−86.69 ± 0.92	−86.53 ± 0.87	WT, *n* = 17; KO, *n* = 14	Unpaired *t* test	*p* = 0.9013
Input resistance (MΩ)	279.70 ± 16.53	273.10 ± 17.32	WT, *n* = 16; KO, *n* = 14	Unpaired *t* test	*p* = 0.7870
4 Hz cumulative frequency (Hz)	5.50 ± 0.54	6.96 ± 0.95	WT, *n* = 10; KO, *n* = 5	Unpaired *t* test	*p* = 0.1744
4 Hz number of spikes	5.20 ± 0.44	6.20 ± 0.86	WT, *n* = 10; KO, *n* = 5	Unpaired *t* test	*p* = 0.2683
8 Hz cumulative frequency (Hz)	6.34 ± 0.61	7.96 ± 0.11	WT, *n* = 11; KO, *n* = 5	Unpaired *t* test	*p* = 0.0994
8 Hz number of spikes	6.18 ± 0.55	6.60 ± 0.40	WT, *n* = 11; KO, *n* = 5	Unpaired *t* test	*p* = 0.6400
Resonance frequency (Hz)	0.900 ± 0.039	0.811 ± 0.0815	WT, *n* = 10; KO, *n* = 6	Unpaired *t* test	*p* = 0.2841
Resonance strength	1.146 ± 0.039	1.266 ± 0.089	WT, *n* = 10; KO, *n* = 6	Unpaired *t* test	*p* = 0.1814
Maximum impedance magnitude (MΩ)	515.9 ± 39.19	556.6 ± 88.17	WT, *n* = 10; KO, *n* = 6	Unpaired *t* test	*p* = 0.6359

Summary of statistical analysis.

### Drugs

The pan-T–type channel blocker *Z*944 (Tringham et al., 2012) was dissolved in DMSO for a final concentration of 0.01%. Stock solutions of Z944 were stored at −20°C and bath applied for 10 min prior to recording. Tetrodotoxin (TTX) was purchased from Alomone Labs (catalog #T-550), and all other chemicals were ordered from Sigma-Aldrich unless specified.

## Results

### Morphological and electrophysiological classification of GC maturation

To examine the contribution of Cav3.2 channels to DG excitability, we used whole-cell patch–clamp recordings from neurons located throughout the DG GCL. As developmental neurogenesis is similar to adult neurogenesis, albeit progressing at a faster rate (Espósito et al., 2005; Laplagne et al., 2006; Zhao et al., 2006; Overstreet-Wadiche et al., 2006a), we used juvenile mice given that the heterogeneity in GC maturational stages is higher compared with adult rodents while still retaining mature “adult-like” GCs. In response to depolarizing current steps, cells exhibited responses that ranged from outward rectification to repetitive spiking (Fig. 1), consistent with the maturational heterogeneity present in the DG (Ambrogini et al., 2004). Cells with the most immature features such as outward rectification (data not shown), rudimentary spikes, and one or two action potentials were located in the inner GCL near the subgranular zone (Fig. 1*A*), where newly postmitotic GCs are predominately located (Espósito et al., 2005). Outwardly rectifying cells are likely comprised of progenitor cells and have previously been morphological characterized (Filippov et al., 2003; Fukuda et al., 2003; Ambrogini et al., 2004), as such we did not further characterize this infrequently observed subpopulation of cells. Rudimentary spiking cells had high *R*_o_ (6.34 ± 1.3 GΩ *n* = 8 pooled WT and KO) and expressed doublecortin (DCX), consistent with immature characteristics (Fig. 1*A*). These cells were morphologically heterogeneous, presumably reflecting the dynamic nature of this maturational stage. Cells that fired one or few small amplitude action potentials were also found near the inner GCL (Fig. 1*B*). These likely represent newly postmitotic GCs early in their integration into the circuit (∼2 weeks postmitosis), herein referred to as immature GCs (Schmidt-Hieber et al., 2004; Heigele et al., 2016). GCs in this category had a high *R*_o_ (4.52 ± 0.85 GΩ *n* = 17 pooled WT and KO) and expressed DCX and typically had dendritic processes that extended into the molecular layer (Fig. 1*B*). Occasionally we also found cells with seemingly immature characteristics such as a high *R*_o_ and localization in the inner GCL but fired one large action potential followed by smaller spikes (Fig. 1*C*). We classified these cells as transitional cells, exhibiting similar characteristics to ∼3 weeks postmitosis immature GCs that lack the voltage-gated Na^+^ and K^+^ channel density to support repetitive spiking (Mongiat et al., 2009). GCs belonging to this group were rarely observed and so were not further characterized.

GCs that fired repetitive action potentials in response to prolonged depolarization were found throughout the GCL (Fig. 1*D*,*E*). The *R*_o_ was variable ranging from 129.9 to 1,469.1 MΩ (*n* = 76 pooled WT and KO), indicating that this population was comprised of GCs with varying degrees of maturation (Mongiat et al., 2009; Heigele et al., 2016; Gonzalez et al., 2018). We classified GCs based on their *R*_o_ as intermediate or mature as described in the following sections. We found that intermediate GCs had dendritic arborizations that extended throughout the molecular layer that were considerably more developed than immature GCs (Fig. 1*D*). Similarly, mature GCs also had extensive dendritic arborizations (Fig. 1*E*), consistent with the rapid dendritic growth that reaches nearly steady state within the first 3 weeks postmitosis (Zhao et al., 2006; Sun et al., 2013).

Overall, the firing patterns and morphology observed throughout the GCL are consistent with previous work, allowing us to differentiate between different GC maturational stages based on their electrophysiological properties. Notably, no obvious morphological differences were observed in GCs from WT and Cav3.2 KO mice, indicating that overall maturation processes might not be impacted by loss of Cav3.2 channels.

### Loss of T-type calcium channels impairs LTCS in immature GCs

In immature GCs, action potential firing is typically preceded by a T-type channel mediated low LTCS that boosts action potential generation (Ambrogini et al., 2004; Schmidt-Hieber et al., 2004). Given the high level of expression in the DG (Talley et al., 1999; McKay et al., 2006), we sought to investigate whether Cav3.2 underlies LTCS and how loss of Cav3.2 may impact excitability in this population. We first observed that LTCS were most frequent in cells located in the inner GCL; these cells were classified as immature by their high *R*_o_ (>1 GΩ) and response to depolarizing current injections including rudimentary spikes or action potential firing cells, as well as occasionally outward rectification. In WT and Cav3.2 KO mice, the majority of cells recorded in the inner GCL were immature GCs that fired a single action potential (WT, *n* = 19/28 cells; KO, *n* = 18/29 cells). The proportion of cells belonging to each class was similar in both mice strains (Fig. 2*A*). In WT mice, LTCS were occasionally found in cells with a rudimentary spike (*n* = 1/4 cells) or outward rectification (*n* = 2/5 cells) and frequently found in immature GCs that fired an action potential (*n* = 15/19 cells; Fig. 2*A*,*B*). The fast action potential was sensitive to blockade of voltage-gated Na^+^ channels with TTX (1 µM; Fig. 2*C*), whereas the LTCS was attenuated with T-type channel blocker Z944 (1 µM; *n* = 4; Fig. 2*C*). In Cav3.2 KO mice, LTCS were also predominately found in action potential firing immature GCs. However, the overall proportion of immature GCs with LTCS was significantly reduced (KO *n* = 3/18 cells; Fisher's exact test, *p* = 0.0002; Fig. 2*B*). These results suggest that T-type channel–mediated LTCS are present early postmitosis but have the highest functional expression in immature GCs that fire few action potentials. Furthermore, the T-type channel subtype Cav3.2 appears to underlie LTCS in immature GCs but is not exclusively required, indicating that another membrane conductance(s) likely also contribute to intrinsic excitability at this maturational stage.

### Contribution of maturational stage and firing properties to GC heterogeneity

**Figure 3.**
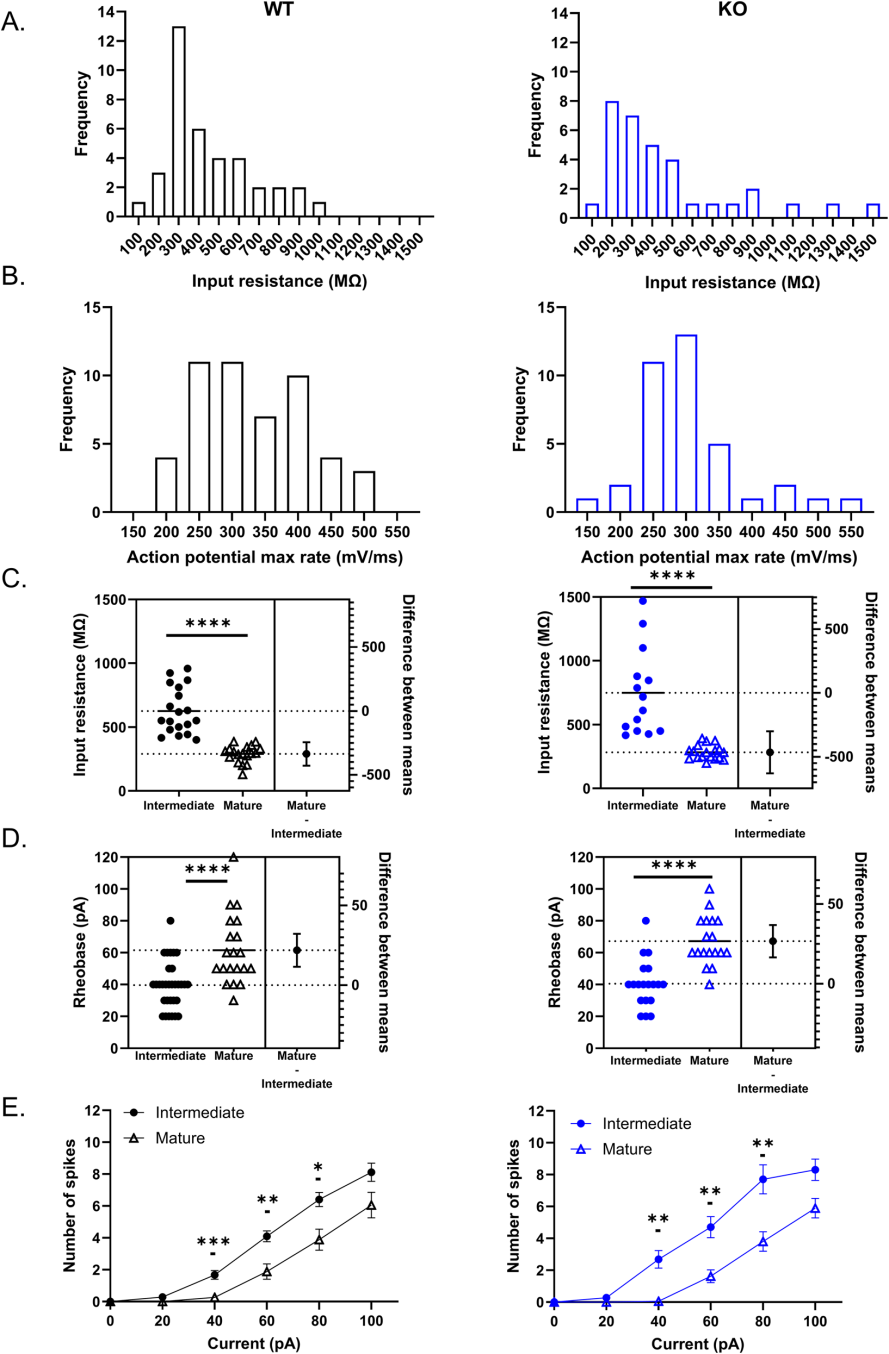
Heterogeneity in intrinsic excitability associated with maturational stages in DG GCs. The wide range of R_o_ (***A***) and action potential rate of rise (***B***) values in GCs from WT (black) and Cav3.2 KO mice (blue) indicates this population of repetitive firing GCs contains varying degrees of maturity. Mature GCs are characterized by a low *R*_o_ (<400 MΩ) and typically have a faster action potential rate of rise (>300 mV/ms), while intermediate GCs have a high *R*_o_ (>400 MΩ) and often have a slower action potential rate of rise. Intermediate GCs were hyperexcitable compared with mature GCs for both WT and Cav3.2 KO mice as evidenced by a significantly higher *R*_o_ (***C***; WT intermediate: 627.1 ± 41.40 MΩ *n* = 19; WT mature: 290.00 ± 15.81 MΩ *n* = 18; unpaired *t* test *p* < 0.0001; KO intermediate: 748.10 ± 90.40 MΩ *n* = 14, KO mature: 283.50 ± 13.19 MΩ *n* = 18; Mann–Whitney test *p* < 0.0001); lower rheobase (***D***; WT intermediate: 39.68 ± 2.68 pA *n* = 31; WT mature: 61.50 ± 4.88 pA *n* = 20; Mann–Whitney test *p* < 0.0001; KO intermediate: 40.53 ± 3.46 pA *n* = 19; KO mature: 67.22 ± 3.60 pA *n* = 18; unpaired *t* test *p* < 0.0001) and firing more action potentials for a given current step (***E***; WT: mixed effects analysis main effect of current *p* < 0.0001; maturation *p* = 0.0010; and interaction *p* < 0.0001; Sidak multiple comparison, 20 pA, *p* = 0.1029; 40 pA *p* = 0.0003; 60 pA *p* = 0.0043; 80 pA *p* = 0.0199 ;and 100 pA *p* = 0.2389; KO: mixed-effect analysis main effect of current *p* < 0.0001; maturation *p* = 0.0001; and interaction *p* < 0.0001. Sidak multiple comparison, 20 pA *p* = 0.5819; 40 pA *p* = 0.0019; 60 pA *p* = 0.0027; 80 pA *p* = 0.0085; 100 pA *p* = 0.076). Overall, the absence of Cav3.2 does not appear to modify the maturation-dependent electrophysiological profile. *p < 0.05; ** *p* < 0.01; *** *p* < 0.001; **** *p* < 0.0001.

We next focused on GCs that fired repetitive spikes in response to depolarizing current injections (Fig. 1*D*,*E*). Throughout GC maturation, *R*_o_ decreases as the expression of channels such as inward rectifying K^+^ channels (Kir), and G-protein–activated inwardly rectifying K^+^ channels is increased (Mongiat et al., 2009; Gonzalez et al., 2018). As such, at ∼4 weeks postmitosis GCs display increased excitability compared with their mature counterparts, firing repetitive action potentials in response to smaller current injections. Consistent with the heterogeneity in maturational stage, we found a wide range of *R*_o_ values in repetitive spiking GCs from both WT and Cav3.2 KO mice (Fig. 3*A*). Based on previous work, we classified GCs with *R*_o_ < 400 MΩ as mature and GCs with *R*_o_ > 400 MΩ as intermediate (Schmidt-Salzmann et al., 2014). The action potential rate of rise also varies with maturation, increasing as GCs mature (Schmidt-Salzmann et al., 2014; Ceanga et al., 2019). We similarly found a broad range of action potential rate of rise values in both WT and Cav3.2 KO, with mature GC values typically >300 mV/ms (Fig. 3*B*). In both WT and Cav3.2 KO mice, intermediate GCs were more excitable than mature GCs (Fig. 3*C*–*E*). The *R*_o_ was significantly higher in intermediate GCs than mature GCs from both WT and KO [WT intermediate: 627.1 ± 41.40 MΩ *n* = 19; WT mature: 290.00 ± 15.81 MΩ *n* = 18; unpaired *t* test *p* < 0.0001; difference between means, −337.1 (95% CI, −428.90 to −245.20); KO intermediate: 748.10 ± 90.40 MΩ *n* = 14; KO mature: 283.50 ± 13.19 MΩ *n* = 18; Mann–Whitney test *p* < 0.0001; difference between means, −464.60 (95% CI: −629.50 to −299.70); Fig. 3*C*], and the rheobase was significantly smaller in intermediate GCs compared with mature GCs in both WT and KO (WT intermediate: 39.68 ± 2.68 pA *n* = 31; WT mature: 61.50 ± 4.88 pA *n* = 20; Mann–Whitney test *p* < 0.0001; difference between means, 21.82 (95% CI, 11.49–32.16); KO intermediate: 40.53 ± 3.46 pA *n* = 19; KO mature: 67.22 ± 3.60 pA *n* = 18; unpaired *t* test *p* < 0.0001; difference between means, 26.22 (95% CI, 16.57–36.83); Fig. 3*D*]. As such, intermediate GCs from both WT and Cav3.2 KO mice fired more action potentials in response to a given current step (Fig. 3*E*; WT: mixed effects analysis main effect of current *p* < 0.0001; maturation *p* = 0.0010 and interaction *p* < 0.0001; Sidak multiple comparison, 20 pA, *p* = 0.1029; 40 pA *p* = 0.0003; 60 pA *p* = 0.0043; 80 pA *p* = 0.0199, and 100 pA *p* = 0.2389; intermediate *n* = 32; mature *n* = 19; KO: mixed-effect analysis main effect of current *p* < 0.0001; maturation *p* = 0.0001; and interaction *p* < 0.0001; Sidak multiple comparison, 20 pA *p* = 0.5819; 40 pA *p* = 0.0019; 60 pA *p* = 0.0027; 80 pA *p* = 0.0085; 100 pA *p* = 0.076; intermediate *n* = 19; mature *n* = 19). Thus, in comparing the electrophysiological properties between WT and Cav3.2 KO GCs, our results suggest that a reduction in excitability with maturation occurs in both genotypes.

**Figure 4.**
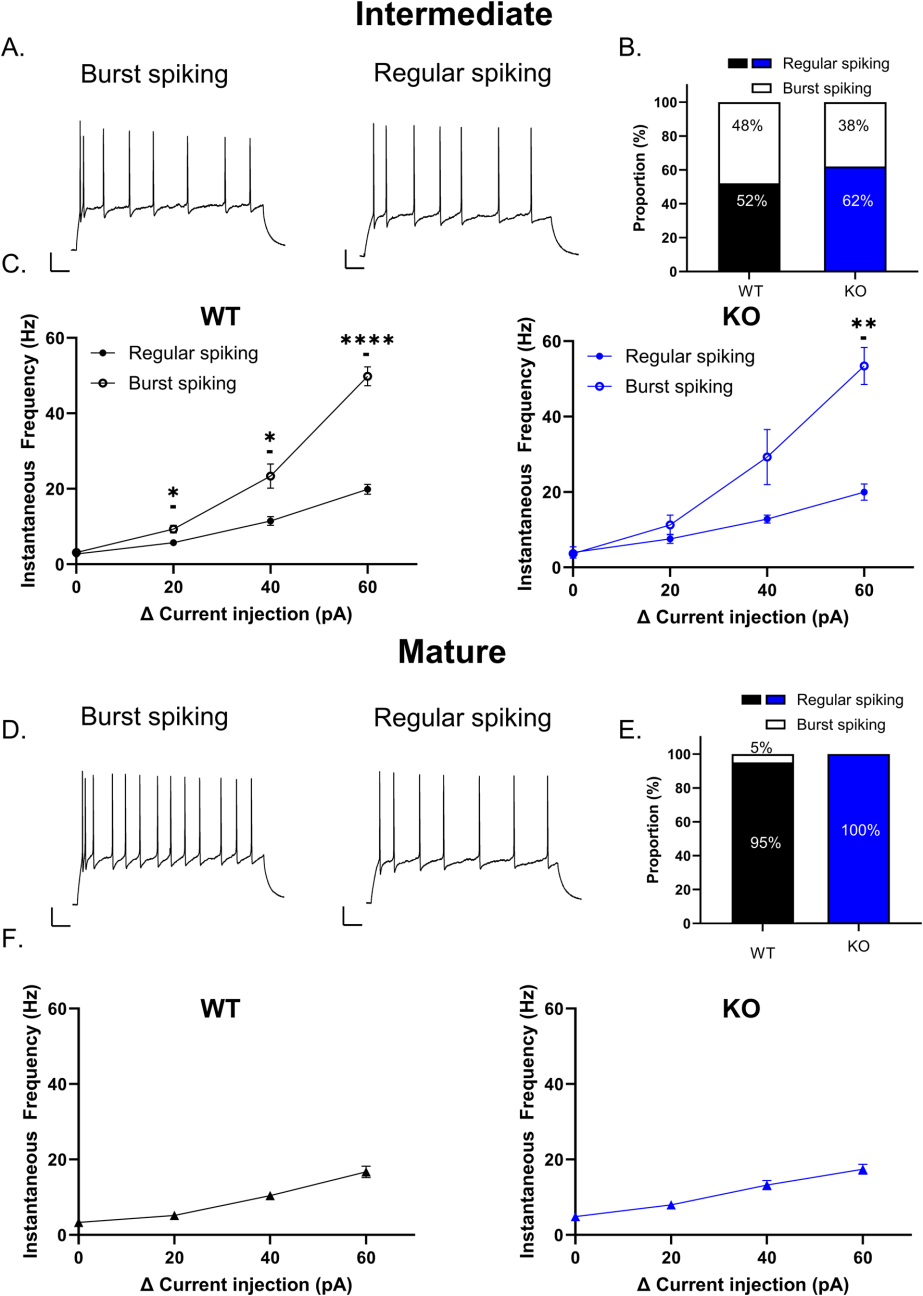
Maturational stage-dependent burst spiking in DG GCs. In response to depolarizing current steps, intermediate maturity DG GCs displayed either a high-frequency initial burst of action potentials (left) or regular spiking (right; ***A***). The proportion of burst spiking cells and regular spiking cells were roughly equivalent in WT. Conversely, regular spiking cells were slightly more predominant in Cav3.2 KO mice (***B***). However, the number of cells belonging to each category was not significantly different (Fisher's exact test *p* = 0.7342). The initial instantaneous firing frequency of the first two action potentials was higher in burst spiking cells compared with regular spiking cells in both WT and Cav3.2 KO (***C***; WT: mixed-effect analysis main effect current *p* < 0.0001; firing pattern *p* < 0.0001; and interaction *p* < 0.0001; Sidak multiple comparison Δ0 pA *p* = 0.9156; Δ20 pA *p* = 0.0299; Δ40 pA *p* = 0.0143 and Δ60 pA *p* < 0.0001; KO: mixed effects analysis main effect current *p* < 0.0001; firing pattern *p* = 0.0029; and interaction *p* < 0.0001; Sidak multiple comparison Δ0 pA *p* = 0.9997; Δ20 pA *p* = 0.6815; Δ40 pA *p* = 0.3045; and Δ60 pA *p* = 0.0041). Conversely, in mature GCs from both WT and Cav3.2 KO, almost all cells displayed regular spiking (***D***, ***E***). The initial instantaneous firing frequency in regular spiking mature GCs is shown in panel ***F***. Scale bar, 100 ms, 20 mV. *p < 0.05; **p < 0.01; ***p < 0.001; ****p < 0.0001.

In addition to maturational stage, heterogeneity in the firing pattern was also observed. In particular, intermediate and mature GCs fired either a short initial burst or regular spiking action potentials (Fig. 4*A*,*D*), similar to previous characterizations (Kim et al., 2023; Kennedy et al., 2024). In intermediate GCs, both burst spiking and regular spiking GCs typically fired two high-frequency action potentials at the onset of depolarizing step, followed by a gradual decrease in frequency firing throughout the rest of the depolarization step. However, the initial instantaneous firing frequency between the first two action potentials was significantly higher in burst spiking cells compared with regular spiking cells with increasing current steps (Fig. 4*C*; WT: mixed effects analysis main effect current *p* < 0.0001; firing pattern *p* < 0.0001 and interaction *p* < 0.0001; Sidak multiple comparison Δ0 pA *p* = 0.9156; Δ20 pA *p* = 0.0299; Δ40 pA *p* = 0.0143; and Δ60 pA *p* < 0.0001; regular spiking *n* = 13; burst spiking *n* = 12; KO: mixed effects analysis main effect current *p* < 0.0001; firing pattern *p* = 0.0029 and interaction *p* < 0.0001; Sidak multiple comparison Δ0 pA *p* = 0.9997; Δ20 pA *p* = 0.6815; Δ40 pA *p* = 0.3045; and Δ60 pA *p* = 0.0041; regular spiking *n* = 8; burst spiking *n* = 5). The proportion of burst spiking and regular spiking cells was roughly equivalent in WT mice, whereas the proportion of burst spiking cells was slightly smaller in Cav3.2 KO, but not significantly different between genotypes (WT: 52% regular spiking; 48% burst spiking *n* = 25; KO: 62% regular spiking, 38% burst spiking *n* = 13; Fisher's exact test *p* = 0.7342). The initial instantaneous firing frequency at rheobase +60 pA was not significantly different between burst spiking cells in WT and Cav3.2 KO mice (initial firing frequency in WT: 49.81 ± 2.50 *n* = 12; KO: 53.43 ± 4.91 *n* = 5; unpaired *t* test *p* = 0.4775). Conversely, in mature DG GCs, almost all cells were regular spiking in both WT and Cav3.2 KO (WT: 95% regular spiking; 5% burst spiking *n* = 19; KO: 100% regular spiking *n* = 15; Fig. 4*D*–*F*). The initial instantaneous firing frequency rarely reached values >25 Hz (Fig. 4*F*).

The higher proportion of regular spiking cells in mature GCs compared with intermediate GCs is consistent with recent findings that T-type channel–mediated burst spiking is suppressed in mature GCs (Kennedy et al., 2024). While the burst spiking that does occur in mature GCs has previously been attributed to Cav3.2 channels (Dumenieu et al., 2018), how loss of Cav3.2 impacts excitability in regular spiking cells has not yet been examined.

### Loss of Cav3.2 increases GC excitability in regular spiking cells

**Figure 5.**
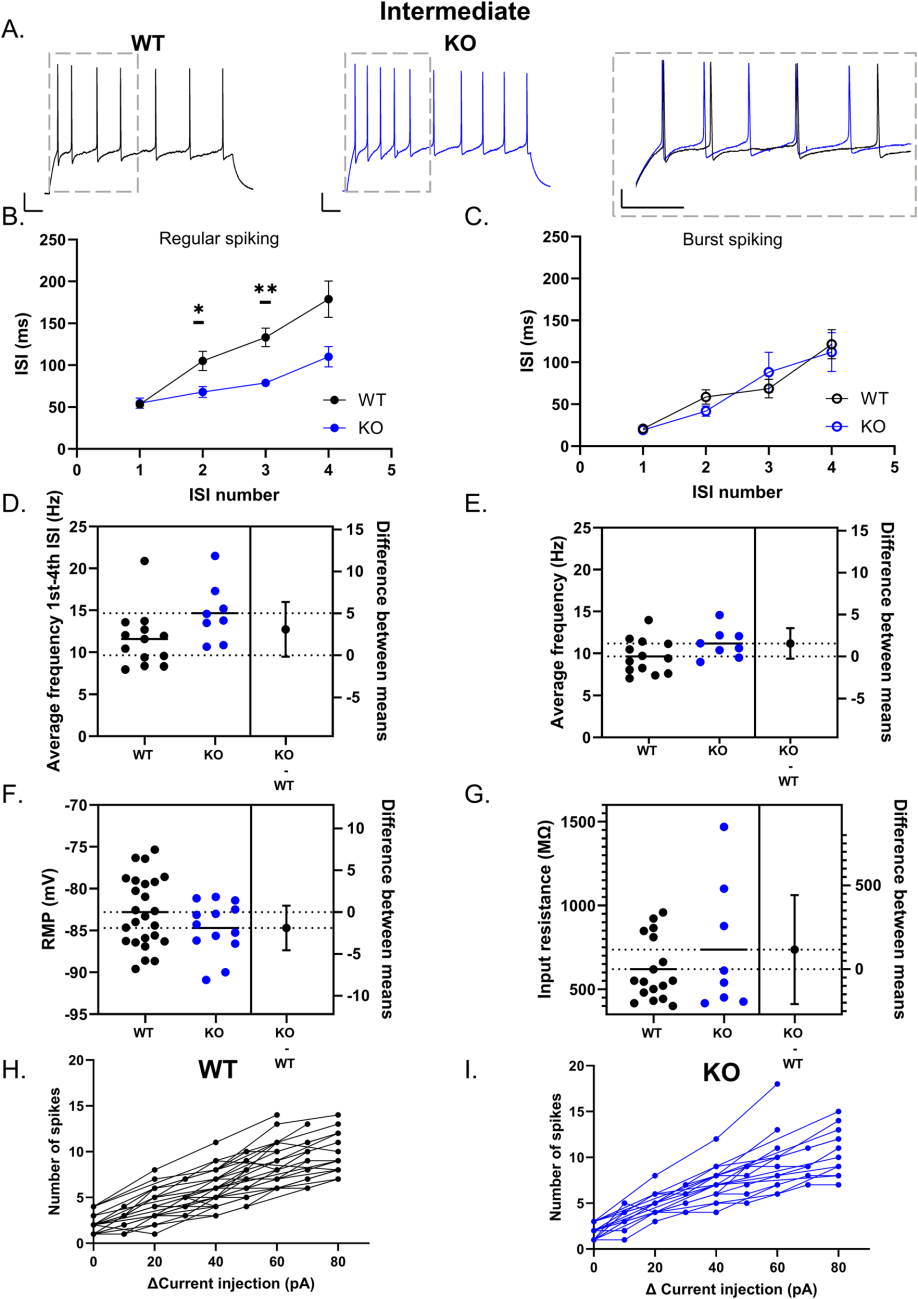
Loss of Cav3.2 increases excitability in intermediate regular spiking GCs. In regular spiking intermediate GCs (***A***, ***B***), the ISI initially increases with successive spikes. The right panel shows magnified region between the first four ISI. The ISI was significantly smaller in GCs from Cav3.2 KO mice compared with WT GCs (2-way ANOVA main effect of ISI number, *p* < 0.0001; genotype, *p* = 0.0033; and interaction, *p* = 0.0253. Sidak multiple comparison 2nd ISI *p* = 0.04575; 3rd ISI *p* = 0.0015; 4th ISI *p* = 0.0526). Conversely, no significant difference was observed in burst spiking cells from WT and Cav3.2 KO mice (***C***). Both the average firing frequency within the first four ISIs (***D***) and total average firing frequency (***E***) were not significantly different between genotypes. The RMP (***F***), *R*_o_ (***G***), and number of spikes per given current step (***H***, ***I***) were not significantly different between intermediate GCs from WT and Cav3.2 KO mice (WT shown in black, KO in blue). Scale bar, 100 ms, 20 mV. *p < 0.05; **p < 0.01.

In order to determine whether Cav3.2 channels shape DG GC excitability in regular spiking cells, we characterized how the ISI changes over time throughout a 1-s-long depolarizing step. In regular spiking cells, the ISI was initially small and increased with successive action potentials in both WT and Cav3.2 KO mice (Fig. 5*A*,*B*). However, while in WT mice, the ISI steeply increased with successive spikes, Cav3.2 KO mice had a significantly smaller ISI (two-way ANOVA main effect of ISI number, *p* < 0.0001; genotype, *p* = 0.0033; and interaction, *p* = 0.0253; Sidak multiple-comparison second ISI *p* = 0.04575; third ISI *p* = 0.0015; fourth ISI *p* = 0.0526; WT *n* = 13; KO *n* = 8). This suggests that GCs lacking Cav3.2 may be able to sustain higher-frequency firing. The average firing frequency within the first four ISIs was slightly but nonsignificantly increased in GCs from Cav3.2 KO compared with WT mice (average frequency for first to fourth ISI WT: 11.57 ± 0.95 Hz *n* = 13; KO: 14.67 ± 1.24 Hz *n* = 8; unpaired *t* test *p* = 0.0614; Fig. 5*D*). Although we compared the ISI across the first five action potentials in order to group cells together, GCs often fired more action potentials throughout the step, and so we used the total average firing frequency to compare overall excitability. The total average firing frequency was also not significantly different in GCs from Cav3.2 KO compared with WT mice (average frequency WT: 9.64 ± 0.56 Hz *n* = 13; KO: 11.18 ± 0.62 Hz *n* = 8; unpaired *t* test *p* = 0.0925; Fig. 5*E*). Conversely, in burst spiking GCs, the initial ISI was small and similarly increased in WT and Cav3.2 KO GCs (Fig. 5*C*). As T-type channels rapidly inactivate at depolarized membrane potentials, different firing patterns can be observed in cells with high T-type channel expression depending on the RMP (Llinás and Jahnsen, 1982). To rule this out, we compared RMP between WT and Cav3.2 KO GCs and found no significant difference (Fig. 5*F*; WT, −82.81 ± 0.83 mV *n* = 25; KO, −84.70 ± 0.88 mV *n* = 13; unpaired *t* test *p* = 0.1607). Furthermore, no significant difference was found in the *R*_o_ indicating that GCs were at a comparable maturational stage (WT: 619.80 ± 45.83 MΩ *n* = 17; KO: 736.70 ± 135.1 MΩ *n* = 8; Mann–Whitney test *p* = 0.7975; Fig. 5*G*). Within all intermediate GCs, no significant difference was observed in the number of spikes per current injection (mixed-effect analysis main effect current *p* < 0.0001; genotype *p* = 0.0737; and interaction *p* = 0.2959; Sidak multiple comparison no significant differences at any current values; WT *n* = 32; KO *n* = 19; Fig. 5*H*,*I*).

**Figure 6.**
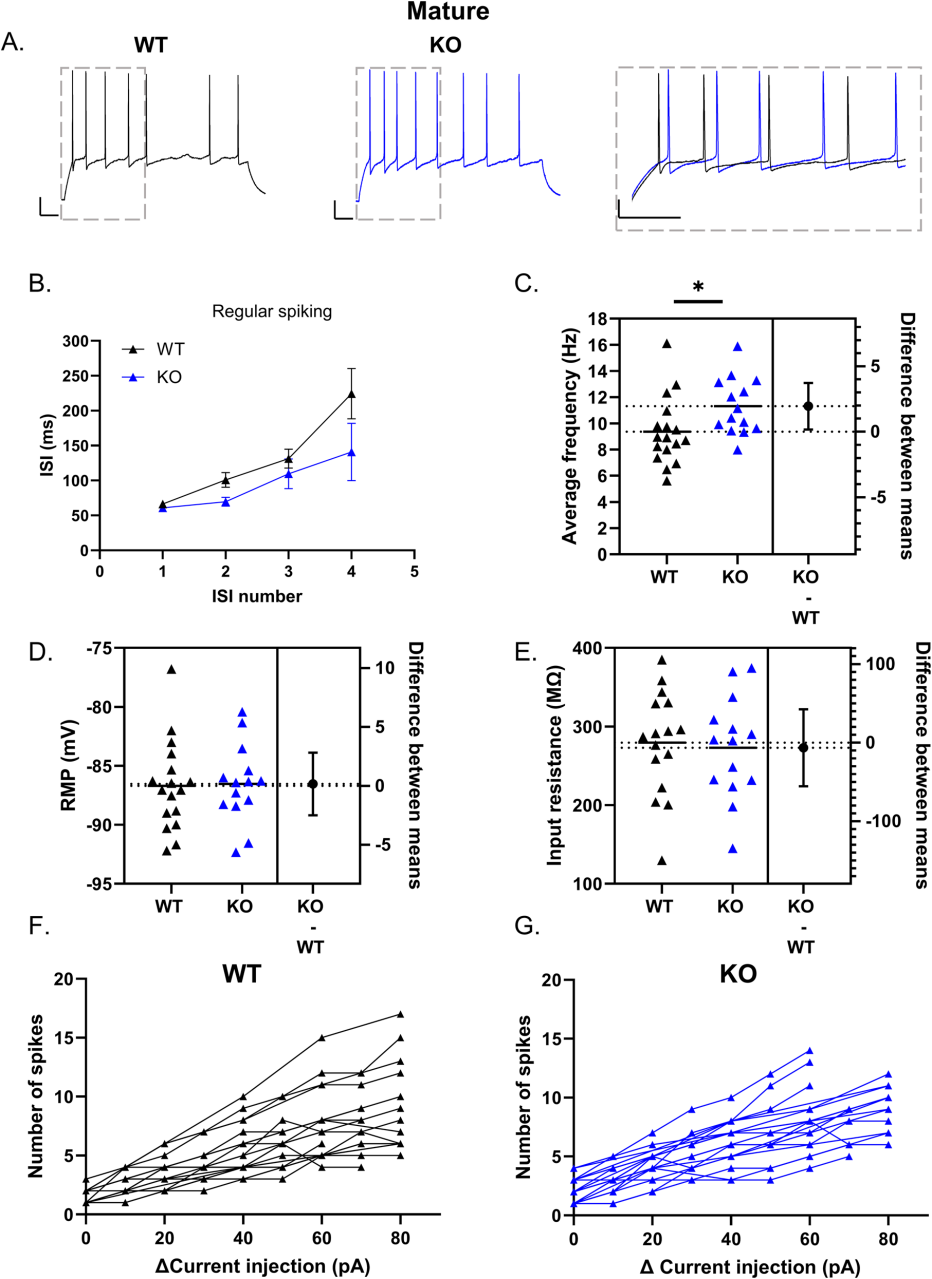
Genetic deletion of Cav3.2 increases firing frequency in mature regular spiking GCs. In regular spiking mature GCs, the initial ISI was similar for GCs from both WT and Cav3.2 KO mice (***A***, ***B***). Despite this, the average frequency throughout the 1-s-long step was slightly higher in GCs from Cav3.2 KO mice compared with WT (***C***) (WT: 9.36 ± 0.63 Hz *n* = 17; KO: 11.32 ± 0.58 Hz *n* = 14; Mann–Whitney test *p* = 0.0131). RMP (***D***), *R*_o_ (***E***), or the number of spikes between mature GCs from WT and Cav3.2 KO mice (***F***, ***G***) was not significantly different. Scale bar, 100 ms, 20 mV. *p < 0.05.

In regular spiking mature DG GCs, ISI also increased throughout action potential trains (Fig. 6*A*,*B*). However, no significant difference was found between WT and Cav3.2 KO mice (two-way ANOVA main effect of ISI, *p* = 0.0002; genotype, *p* = 0.0726; interaction, *p* = 0.2309; WT *n* = 17; KO *n* = 14). Nonetheless, the average firing frequency for all action potentials within the train was slightly but significantly higher in Cav3.2 KO compared with WT [WT: 9.36 ± 0.63 Hz *n* = 17; KO: 11.32 ± 0.58 Hz *n* = 14; Mann–Whitney test *p* = 0.0131; difference between means, 1.96 (95% CI, 0.18 to 3.73); Fig. 6*C*]. This difference is likely not apparent in the ISI plots given that we limited our analysis to the first five action potentials, indicating that the difference appears later in the action potential train. No significant difference was observed in the RMP or *R*_o_ between WT and Cav3.2 KO mice (RMP WT: −86.69 ± 0.92 mV *n* = 17; KO: −86.53 ± 0.87 mV *n* = 14; unpaired *t* test *p* = 0.9013; *R*_o_ WT: 279.70 ± 16.53 *n* = 16; KO: 273.10 ± 17.32 *n* = 14; unpaired *t* test *p* = 0.7870, respectively; Fig. 6*D*,*E*). The total number of action potentials per current injection steps was also not significantly different (Fig. 6*F*,*G*; mixed-effect analysis main effect current *p* < 0.0001; genotype *p* = 0.2227; and interaction *p* = 0.9019; WT: *n* = 20; KO: *n* = 19).

Overall, these results indicate that loss of Cav3.2 channels in regular spiking GCs increases excitability. For intermediate GCs, Cav3.2 KO neurons do not undergo a similar increase in ISI throughout a train of action potentials, while in mature GCs, loss of Cav3.2 allows GCs to fire at an overall higher frequency.

### Increased excitability in Cav3.2 KO does not impact fidelity of frequency-dependent responses

**Figure 7.**
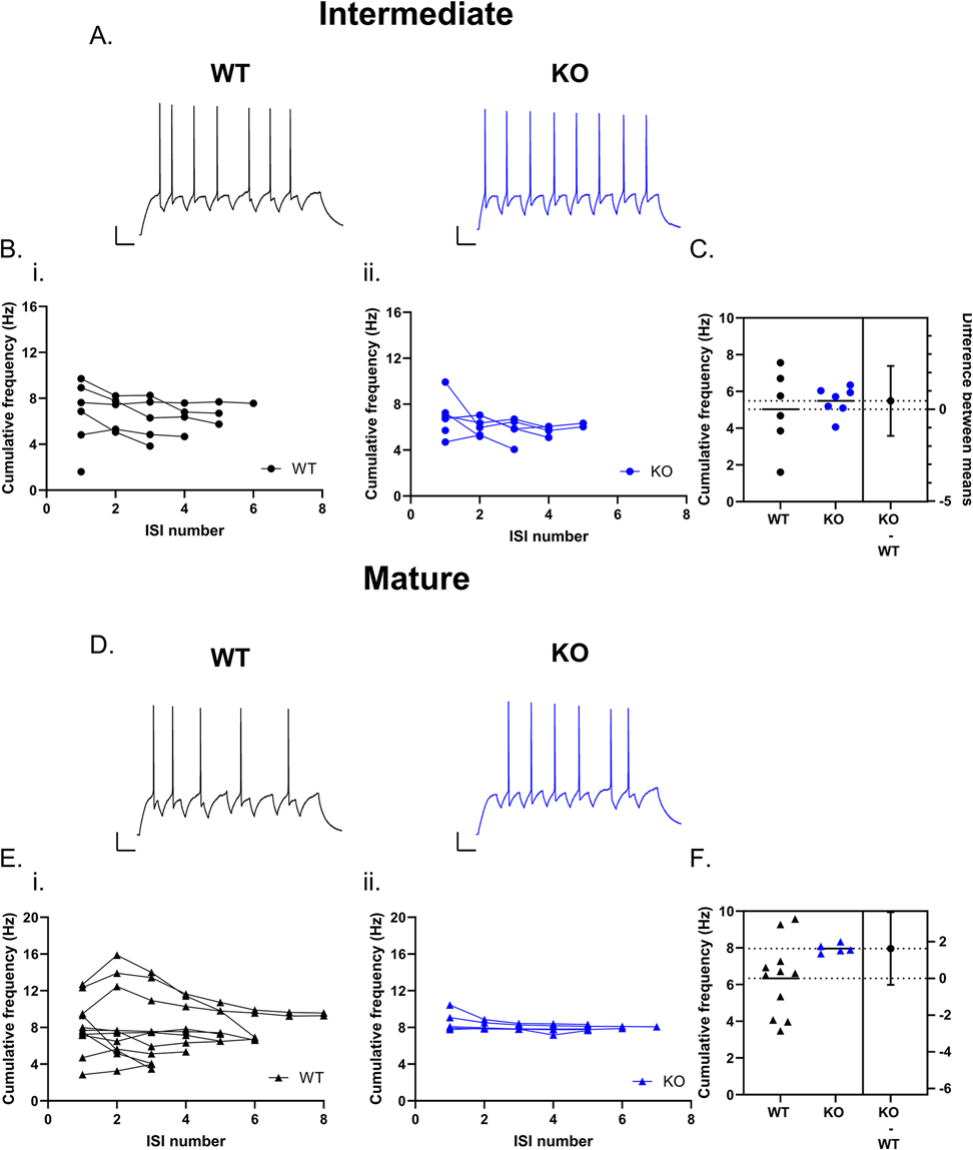
Loss of Cav3.2 does not impact suprathreshold frequency-dependent responses in regular spiking GCs. Intermediate (***A–C***) and mature (***D–F***) regular spiking GCs were injected with suprathreshold current at a frequency of 8 Hz to study frequency-dependent responses. Example traces show the response to 8 Hz frequency stimulation in intermediate (***A***) and mature GCs (***D***). The cumulative frequency across ISI number for each Individual cell is shown in panel ***B*** for intermediate GCs from WT (***i***) and Cav3.2 KO (***ii***) mice. Loss of Cav3.2 did not significantly impact the average cumulative firing frequency in intermediate GCs (***C***). The cumulative frequency across the ISI number for each mature GC is shown in panel ***E*** for WT (***i***) and Cav3.2 KO (***ii***) mice. The average cumulative firing frequency was not significantly different in mature GCs between genotypes (***F***). Scale bar, 100 ms, 20 mV.

The hippocampus is part of an oscillatory network that exhibits theta rhythms, sharp wave-ripple complex, and gamma rhythms during specific behaviors (Colgin, 2016). In the DG, action potentials are phase locked to theta–gamma oscillations (Pernía-Andrade and Jonas, 2014). As T-type channels can promote oscillatory activity and resonance in other brain regions (Hutcheon et al., 1994; Hughes et al., 2002; Matsumoto-Makidono et al., 2016), we examined whether Cav3.2 channels also shape GC responses to suprathreshold and subthreshold oscillatory activity in regular spiking GCs. For suprathreshold stimulation, we stimulated at an intensity 1.5× rheobase, which consistently elicited action potential firing at the input frequency used. At 4 Hz, intermediate regular spiking GCs from WT and KO mice were able to follow 4 Hz input frequency (cumulative frequency WT: 3.85 ± 0.39 Hz *n* = 6; KO: 4.58 ± 0.25 Hz *n* = 8; unpaired *t* test *p* = 0.1223) The number of action potentials was not significantly different between WT and KO (WT: 3.67 ± 0.33 spikes *n* = 6; KO: 4.25 ± 0.25 spikes *n* = 8; unpaired *t* test *p* = 0.1779; data not shown). At 8 Hz, the average cumulative frequency for both WT and Cav3.2 KO intermediate regular spiking GCs was slightly lower than the input stimulation, but did not differ between genotypes (WT: 5.02 ± 0.87 Hz *n* = 6; KO: 5.48 ± 0.29 Hz *n* = 7; unpaired *t* test *p* = 0.6063; Fig. 7*A*–*C*). The number of action potentials was also not significantly different between WT and KO (WT: 5.00 ± 0.73 *n* = 6; KO: 4.43 ± 0.57 *n* = 7; unpaired *t* test *p* = 0.5448). Overall, these results indicate that for intermediate regular spiking GCs, loss of Cav3.2 channels does not impact their ability to follow input frequencies.

In mature GCs, WT and Cav3.2 KO regular spiking cells responded similarly to the 4 Hz input stimulation reaching mean cumulative frequencies slightly higher than input 4 Hz frequency (WT: 5.50 ± 0.54 Hz *n* = 10; KO: 6.96 ± 0.95 Hz *n* = 5; unpaired *t* test *p* = 0.1744). Furthermore, the total number of action potentials during the stimulation was also not significantly impacted by genotype (WT: 5.20 ± 0.44 spikes *n* = 10; KO: 6.20 ± 0.86 spikes *n* = 5; unpaired *t* test *p* = 0.2683; data not shown). At 8 Hz stimulation frequency, the average cumulative frequency was not significantly different between genotypes (cumulative frequency WT: 6.34 ± 0.61 Hz *n* = 11; KO: 7.96 ± 0.11 Hz *n* = 5; unpaired *t* test *p* = 0.0994; Fig. 7*E*), and the number of action potentials fired during stimulation was also similar between genotypes (WT: 6.18 ± 0.55 spikes *n* = 11; KO: 6.60 ± 0.40 spikes *n* = 5; unpaired *t* test *p* = 0.6400). Thus, in regular spiking GCs, loss of Cav3.2 does not substantially impact the ability to follow input frequencies despite leading to higher-frequency firing in prolonged depolarization steps in intermediate and mature GCs.

**Figure 8.**
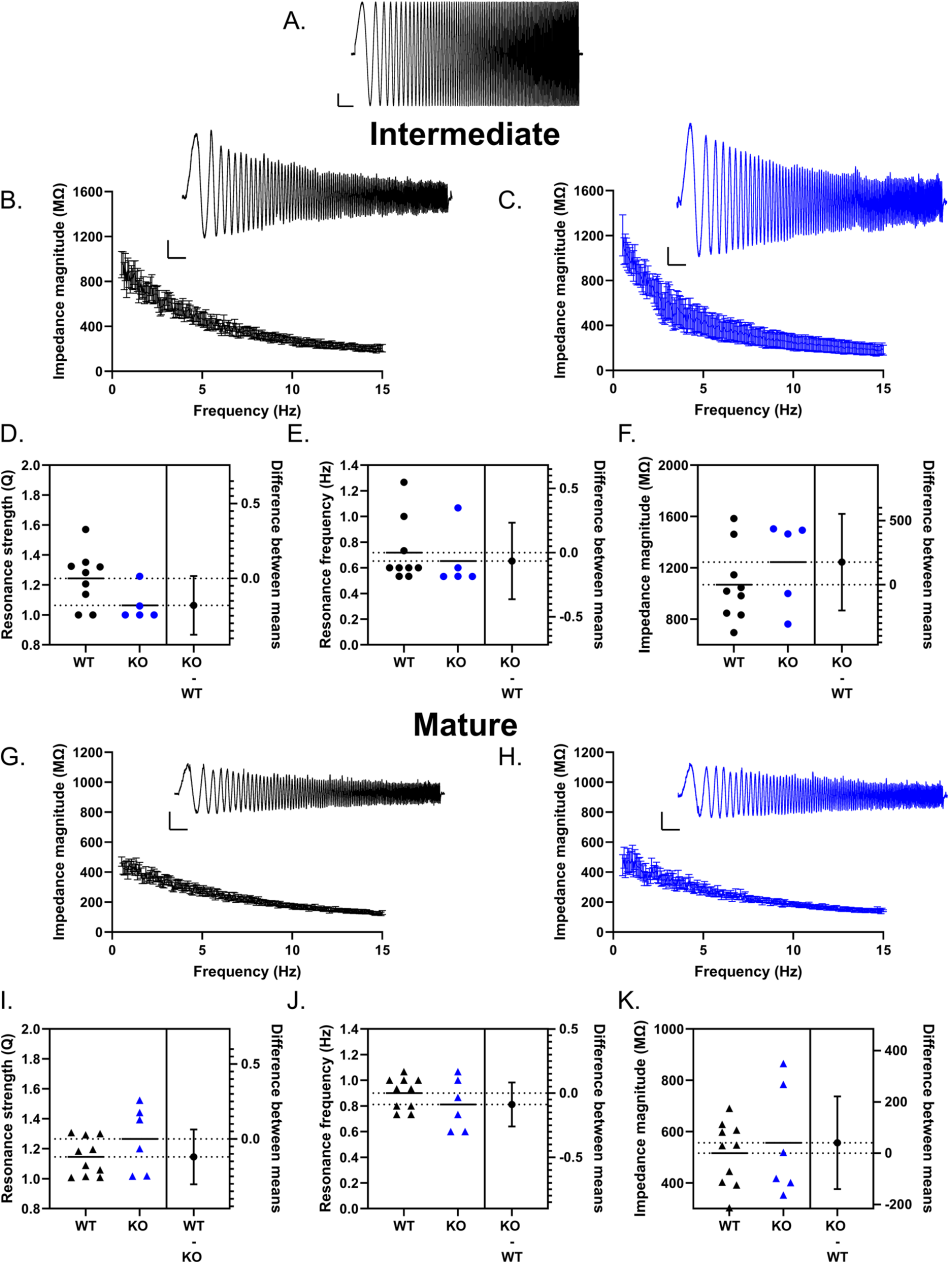
Subthreshold frequency-dependent response of GCs is not impacted by loss of Cav3.2. Subthreshold frequency-dependent responses were studied using a CHIRP stimulus (0–15 Hz in 15 s; ***A***). In both intermediate (***B***, ***C***) and mature DG GCs (***G***, ***H***), no resonance peak was observed in the impedance magnitude profile (impedance magnitude profile shows mean with SEM). Instead, high-frequency responses were strongly attenuated, indicating that GCs behaved as a low-pass filter. Consistent with a lack of resonance, the resonance strength values were near 1 for both WT and Cav3.2 KO intermediate (***D***) and mature GCs (***I***), and the resonance frequency was <1 Hz for both genotypes (intermediate GCs panel ***E***, mature GCs panel ***J***). The maximum impedance magnitude was not significantly impacted by loss of Cav3.2 in both intermediate (***F***) and mature (***K***) GCs. CHIRP waveform scale bar, 5 pA, 1 s. GC response, 5 mV, 1 s.

In addition to mediating suprathreshold excitability, the low-threshold nature of T-type channels enables them to shape subthreshold properties such as resonance (Hutcheon et al., 1994). In the hippocampus, the intrinsic properties of principal cells and interneurons can support resonance, such as CA1 pyramidal cells that show resonance around theta (∼2–7 Hz; Pike et al., 2000; Hu et al., 2002). This subthreshold resonance can also drive spike frequency preference, whereby action potential firing is promoted at frequencies near resonance peak and attenuated at other frequencies (Hutcheon et al., 1996; Pike et al., 2000). In order to study resonance, we used a CHIRP stimulation (0–15 Hz in 15 s) to characterize the ZAP (Fig. 8; Hu et al., 2002; Mishra and Narayanan, 2020). In general, membrane resonance is evidenced by a maximum peak in the voltage response; ZAP analysis of both intermediate and mature GCs of either genotype did not show any peak, indicating lack of resonance. Rather, GC responses to high-frequency stimulation were strongly attenuated, behaving as a low-pass filter with maximal impedance frequencies <1 Hz (Fig. 8*B*,*C*,*E*,*G*,*H*,*J*) consistent with previous work in mature DG GCs (Krueppel et al., 2011; Mishra and Narayanan, 2020). In both genotypes, the maximum impedance magnitude was higher in intermediate compared with mature GCs, as expected for a cell with a higher *R*_o_ [intermediate WT: 1,068 ± 97.22 MΩ *n* = 9; mature WT: 515.9 ± 39.19 MΩ *n* = 10; unpaired *t* test *p* < 0.0001 difference between means, −552.2 (95% CI −764.90 to −339.40); intermediate KO: 1,244 ± 153.4 MΩ *n* = 5; mature KO: 556.6 ± 88.17 MΩ *n* = 6; unpaired *t* test *p* = 0.0028; difference between means, −687.80 (95% CI −1,071 to −304.80)]. When comparing genotypes, no significant differences were found between WT and KO intermediate or mature GCs for the resonance frequency (intermediate WT: 0.72 ± 0.083 Hz *n* = 9; intermediate KO: 0.653 ± 0.104 Hz *n* = 5; Mann–Whitney test *p* = 0.2118; mature WT: 0.900 ± 0.039 Hz *n* = 10; mature KO: 0.811 ± 0.0815 Hz *n* = 6; unpaired *t* test *p* = 0.2841). However, in intermediate GCs, the resonance strength (*Q*) calculated as the ratio of the maximum impedance amplitude to the impedance amplitude at 0.5 Hz was slightly but nonsignificantly higher in WT compared with KO (WT: 1.24 ± 0.060 *n* = 9; KO: 1.06 ± 0.050 *n* = 5; unpaired *t* test *p* = 0.0686; Fig. 8*D*). Given that intermediate GCs do not show any resonance, the functional relevance of this slight difference in *Q* values is unclear, potentially reflecting the ability of WT GCs to sustain larger initial voltage responses prior to the rapid attenuation. This was not significantly different in mature DG GCs (WT: 1.146 ± 0.039 *n* = 10; KO: 1.266 ± 0.089 *n* = 6 unpaired *t* test *p* = 0.1814; Fig. 8*I*), indicating that it appears to be specific to intermediate GCs. Lastly, no significant difference was found in the maximal impedance magnitude between genotypes for each maturational stage (intermediate WT: 1,068 ± 97.22 MΩ *n* = 9; intermediate KO: 1,244 ± 153.4 MΩ *n* = 5 unpaired *t* test *p* = 0.3275; mature WT: 515.9 ± 39.19 MΩ *n* = 10; mature KO: 556.6 ± 88.17 MΩ *n* = 6 unpaired *t* test *p* = 0.6359; Fig. 8*F*,*K*).

Overall, our data indicate that loss of Cav3.2 channels does not substantially impact frequency-dependent responses in the suprathreshold or subthreshold range for either maturational stage. However, previous in vivo studies have found that hippocampal oscillations are impacted in Cav3.2 KO mice (Dumenieu et al., 2018; Arshaad et al., 2021). Thus, while the intrinsic oscillatory properties of GCs do not appear to be altered, loss of Cav3.2 may instead alter network oscillatory properties.

## Discussion

The maturation and integration of GCs into the DG circuit occurs over several weeks, during which GC morphology as well as the density of ion channels develops (Espósito et al., 2005; Mongiat et al., 2009; Heigele et al., 2016). Across different brain regions, the effect of T-type channels on excitability depends on their somatodendritic distribution and coupling to other voltage- and/or Ca^2+^-activated conductances (Wolfart and Roeper, 2002; Crandall et al., 2010; Engbers et al., 2012; Dumenieu et al., 2018). In this study, we frequently observed LTCS in immature GCs in response to small membrane depolarization that preceded the threshold for action potential generation. We found that the proportion of immature GCs displaying LTCS was significantly reduced in Cav3.2 KO mice, indicating a contribution of Cav3.2 channels to excitability at early stages of GC maturation through LTCS generation (Fig. 2*B*). The high *R*_o_, LTCS, and limited action potential firing observed in this population are consistent with GCs aged <3 weeks old (Schmidt-Hieber et al., 2004; Couillard-Despres et al., 2006; Heigele et al., 2016). During this period, synaptic activity is largely mediated by depolarization from GABAergic interneurons (Espósito et al., 2005; Overstreet-Wadiche et al., 2005; Markwardt et al., 2011, 2009; Vaden et al., 2020), promoting synaptic unsilencing (Chancey et al., 2013), action potential firing (Heigele et al., 2016), as well as dendritic outgrowth and integration of GCs in the DG (Ge et al., 2006). The low threshold for activation of T-type channel–mediated LTCS, around −56 mV (Schmidt-Hieber et al., 2004), is well suited for activation by GABAergic depolarization (Ge et al., 2006). As T-type channels can also provide a source of intracellular calcium that regulate neuronal processes such as proliferation, differentiation, and maturation (Chemin et al., 2002b; Lory et al., 2006; Yabuki et al., 2021), calcium influx via Cav3.2 channels may promote GC maturation and integration. While we did not study integration per se, our results indicate that electrophysiological maturation appears to be unaffected by loss of Cav3.2 given that GCs displayed normal maturational-dependent changes in excitability (Fig. 3).

Within individual neurons, all three T-type channels can be expressed to various degrees depending on the brain region (McKay et al., 2006). In regions where the expression of one T-type channel subtype is predominant, subtype-specific KO completely abolishes T-type channel–mediated firing patterns (Kim et al., 2001). Conversely, in neurons expressing more than one T-type channel subtype, KO of multiple subtypes can be required for complete loss (Lee et al., 2014; Pellegrini et al., 2016). In this study, although the proportion of immature DG GCs with LTCS was substantially reduced with Cav3.2 KO, LTCS remained present in ∼17% of cells (Fig. 2*B*), indicating that Cav3.2 channels may not solely underlie LTCS. In addition to Cav3.2, other voltage-gated calcium channels operating in a similar voltage range such as Cav3.1, Cav3.3, or Cav2.3 may also contribute to LTCS. In particular, Cav3.1 channels are a likely candidate for contributing to LTCS in immature GCs as they are shown to be expressed in DCX-positive cells of the DG (Yabuki et al., 2021). Alternatively, LTCS observed in Cav3.2 KO neurons may result from compensatory upregulation of a different low-voltage–activated calcium channel. While we cannot currently rule this out, a recent study using Cav3.2 KO mice did not find compensatory changes in the transcription of either Cav3.1 or Cav3.3 (Arshaad et al., 2021). Notably, given that our experimental approach inherently includes a wide range of postmitotic stages, it is possible that Cav3.1 and Cav3.2 channels mediate LTCS at particular maturational stages before Cav3.2 channels predominate later in maturation. Future studies that focus on narrow ranges of maturation will be required to address this issue. Despite these limitations, the reduced proportion of GCs with LTCS in Cav3.2 KO mice indicates that Cav3.2 channels are an important mediator of this characteristic electrophysiological feature in immature GCs.

As GCs mature, LTCS were no longer readily observed and were virtually absent in intermediate GCs, indicating there exists a critical window for LTCS during GC maturation. This restricted window could be the result of a maturational-dependent downregulation of T-type channel expression. During brain development, T-type channel expression is dynamic. For instance, in the rodent hippocampus, Cav3.2 expression initially increases from P0 to P21 then decreases at P60 (Aguado et al., 2016). Although a similar trend could occur during GC maturation, the strong expression of Cav3.2 in DG GCs (Talley et al., 1999; McKay et al., 2006; Bernal Sierra et al., 2017) indicates that Cav3.2 remains highly expressed in mature GCs. Instead, the restricted window for LTCS likely arises from maturational changes in the morphological and electrophysiological properties that create a permissive period for LTCS. In newborn GCs, dendritic processes are initially short (Fig. 1*A*–*C*) and then rapidly develop during the first 3 weeks to reach the cone-shaped arbor seen in mature GCs (Zhao et al., 2006; Sun et al., 2013; Fig. 1*D*,*E*). Furthermore, immature GCs possess a small membrane capacitance and high *R*_o_ making them electronically compact (Heigele et al., 2016). These features would inherently promote larger depolarization throughout immature GC compartments, enhancing the recruitment of T-type calcium channels. Furthermore, in mature GCs, voltage propagation is strongly attenuated from the dendrites to the soma (Krueppel et al., 2011) as a consequence of both the basic cable properties and the high density of dendritic A-type K^+^ channels (Kim et al., 2018; Oulé et al., 2021). Although the subcellular distribution of Cav3.2 channels is somewhat controversial, with reports indicating the highest Cav3.2 expression in the dendrites (Martinello et al., 2015) or soma (McKay et al., 2006) and electrophysiological evidence pointing toward the axon initial segment (Martinello et al., 2015; Dumenieu et al., 2018), the combined morphological properties and lower-voltage–gated K^+^ channel expression in immature GCs (Mongiat et al., 2009) may promote T-type channel recruitment by somatic depolarization that would otherwise be attenuated in mature GCs. In support of this notion, blocking voltage-gated Na^+^ and K^+^ channels in mature GCs uncovers a small transient calcium spike (Blaxter et al., 1989), indicating that T-type channels remain functionally present but are masked by other conductances in somatic recordings. Thus, the combined low expression of opposing conductances and permissive morphological properties that support LTCS in immature GCs appear to be lost with maturation.

In intermediate and mature GCs, the frequency of action potentials increased throughout an action potential train (Fig. 4*C*,*F*). However, in a subpopulation of GCs, the initial instantaneous firing frequency was significantly higher, firing a short high-frequency burst of action potentials prior to spike frequency adaptation (Fig. 4*A*). In both genotypes, burst spiking was frequently observed in intermediate GCs but was nearly completely absent in mature GCs (Fig. 4*B*,*E*). Previous studies in DG GCs have indicated that burst firing appears to depend on several factors including the dorsal–ventral distribution (Kim et al., 2023), intracellular signaling pathways, and maturational stage (Kennedy et al., 2024). Although we did not investigate the dorsal–ventral distribution, our results are consistent with a maturational stage-dependent contribution of burst spiking, decreasing with GC maturation. In WT intermediate GCs, the proportion of burst spiking and regular spiking cells was roughly ∼48%, while in Cav3.2 KO intermediate GCs, burst spiking cells were slightly less frequently observed (∼38% burst spiking; Fig. 4*B*). This reduction in the proportion of burst spiking GCs suggests that Cav3.2 channels contribute to the initial burst in intermediate GCs as previously described for mature GCs (Dumenieu et al., 2018). Yet, the presence of burst spiking cells in Cav3.2 KO mice indicates that bursts can still occur in younger GCs in the absence of Cav3.2 channels.

In regular spiking GCs, loss of Cav3.2 increased excitability as evidence by the shorter interval between spikes throughout the action potential train in intermediate GCs (Fig. 5*B*) and increased total firing frequency in mature GCs (Fig. 6*C*). A potential mechanism underlying this effect could involve the coupling of Cav3.2 channels to channels involved in setting the firing frequency such as Ca^2+^-activated K^+^ channels. In other brain regions, T-type channels have been shown functionally coupled to Ca^2+^-activated K^+^ channels as well A-type K channels via K^+^ channel-interacting proteins (Wolfart and Roeper, 2002; Molineux et al., 2005; Engbers et al., 2012; Anderson et al., 2010, 2013; Turner and Zamponi, 2014). The small conductance (SK), intermediate conductance (IK), and big conductance (BK) channels are all expressed to variable degrees in DG GCs (Sailer et al., 2004, 2006; Turner et al., 2015), and the functional role of SK channels (Mateos-Aparicio et al., 2014) as well as the indirect role of BK channels (Brenner et al., 2005) in setting GC firing frequency has been shown. An increase in excitability occurring with loss or blockade of T-type channels is also consistent with previous work in mature DG GCs (Dumenieu et al., 2018, Figure 4; Laker et al., 2021). Thus, in regular spiking GCs, Cav3.2 channels may couple to a Ca^2+^-activated K^+^ channel, such that activation of Cav3.2 channels reduces excitability by regulating the interval between spikes. Notably, given that GCs lack Cav3.2 channel–mediated calcium influx throughout their maturation, we cannot rule out that other maturational-dependent mechanisms may also be occurring. Future studies will be required to investigate the underlying mechanism.

Neuronal resonance occurs as a result of interaction between those membrane properties that attenuate high frequencies and voltage-dependent conductances that actively oppose lower frequencies (Hutcheon and Yarom, 2000). The overlap between these two components forms a band pass filter with a narrow frequency range where neurons optimally respond to inputs. The biophysical properties of T-type channels are conducive to resonance as their slow inactivation relative to the membrane time constant attenuates voltage responses at low frequencies, while fast activation amplifies peak frequencies (Hutcheon et al., 1994). In brain regions with high T-type channel expression such as the inferior olive and thalamus, T-type channels are associated with resonance frequencies of ∼4 Hz (Hutcheon et al., 1994; Puil et al., 1994; Matsumoto-Makidono et al., 2016). Although GCs appear to strongly express Cav3.2 channels, our results as well as previous studies have found that mature DG GCs do not show resonance and instead behave as low-pass filters (Fig. 8*G*,*H*; Krueppel et al., 2011; Mishra and Narayanan, 2020). Furthermore, we found that intermediate GCs similarly lack the depolarizing hump indicative of resonance (Fig. 8*B*,*C*), suggesting that the low-pass filter property is established prior to GC maturation. Although the DG is part of an oscillatory network, in particular in the theta and gamma range (Pernía-Andrade and Jonas, 2014), GCs lack the intrinsic properties that promote oscillatory activity. Instead of producing resonance, T-type channels can also function as an amplifying conductance, amplifying resonance mediated by other channels such as hyperpolarization-activated cyclic nucleotide-gated (HCN) channels (Matsumoto-Makidono et al., 2016). While DG GCs express HCN channels, the lack HCN channel-mediated voltage sag and resonance has been suggested to occur as a result of the slow HCN channel kinetics (Mishra and Narayanan, 2020), and the high expression of inwardly rectifying K^+^ currents relative to HCN channel currents (Stegen et al., 2012). Thus, without HCN channel-mediated resonance, Cav3.2 channels appear to amplify only the low frequencies that elicit sufficient membrane depolarization to reach threshold for T-type channel activation.

Previous studies have implicated Cav3.2 channels in hippocampal-mediated behaviors. In particular, Cav3.2 KO mice are impaired in the ability to discriminate between similar inputs (Gangarossa et al., 2014) and contextual learning (Chen et al., 2012) and exhibit attenuated development of temporal lobe epilepsy following pilocarpine treatment (Becker et al., 2008). Despite this strong phenotype, our results indicate that the effects of Cav3.2 KO on GC intrinsic excitability are relatively subtle. In addition to the DG, Cav3.2 channels are also expressed in the entorhinal cortex and other hippocampal subfields (Talley et al., 1999; McKay et al., 2006; Aguado et al., 2016), where functional roles are beginning to emerge (Chen et al., 2012; Topczewska et al., 2023). As a result, which population of neurons in the hippocampus circuit underlies the memory deficits and decreased epileptogenesis observed in Cav3.2 KO mice remains speculative. Nonetheless, our results expand the understanding of how Cav3.2 channels shape the hippocampal circuit.

It is known that neuronal activity results from dynamic interactions of ion channels differentially distributed at distinct membrane compartments. During GC maturation, the complexity of the electrical compartment changes and concomitantly Cav3.2-mediated regulation of excitability evolves from generating LTCS that precedes firing in immature GCs to regulating firing frequency in repetitive spiking intermediate and mature GCs. As gain-of-function mutations in Cav3.2 are linked to neurological disorders including epilepsies (Weiss and Zamponi, 2020), increased calcium influx via Cav3.2 channels has the potential to alter the tightly controlled DG circuit dynamics that allow the DG to function as a robust filter of cortical activity.

## Synthesis

Reviewing Editor: Viji Santhakumar, University of California Riverside Decisions are customarily a result of the Reviewing Editor and the peer reviewers coming together and discussing their recommendations until a consensus is reached. When revisions are invited, a fact-based synthesis statement explaining their decision and outlining what is needed to prepare a revision will be listed below. The following reviewer(s) agreed to reveal their identity: Justin Botterill, Joseph Glykys.

The study examined the role of Cav3.2 in the intrinsic excitability of dentate gyrus granule cell (DGGC) subpopulations at different stages of maturation. The main finding of this detailed study was that knocking out Ca3.2 decreased the proportion of immature DGGC showing low threshold calcium spiles (LTCS). However, they find a subset of immature DGGCs from KO mice that show LTCS, likely mediated by other VGCC which is not explored further. Additionally, the study identifies that intermediate and mature DGGC in Cav3.2 KO mice show an increase in excitability. While limited in scope, the study is meticulous and carefully presented. While limitations are carefully considered in the discussion, there is considerable speculation on the relevance of the findings to epilepsy, learning, and memory which are premature.

1. Given the results showing LTCS in a subset of cells in the KO, the authors should present additional validation of the Cav3.2 KO mice. Although Cav3.2 KO was reported to be confirmed through genotyping, additional characterizations/confirmation to validate Cav3.2 KO in the dentate gyrus through immunostaining would strengthen the study. Previous work using Cav3.2 GFP knock-in mice provided convincing GFP expression that was primarily seen in the granule cell mossy fibers (see Sierra et al., 2017; Frontiers in Molecular Neuroscience, PMID #28360836).

2. There is a missed opportunity in that authors do not follow up on the potential mechanisms of the LTCS in the Cav3.2 KO mice. In Figure 2C, the effect of Z944 was examined only in the WT. Does Z499 block the LTCS in immature DGGCs from Cav3.2 KO mice? Also, he TTX data is not described in the results. On a similar note, why Cav3.2 KO would increase excitability in intermediate and mature DGGCs, while discussed, was not examined.

3. Please clarify whether physiology data was corrected for liquid junctional potential. The methods indicate that the date was not corrected for LJP ~ +14.5 mV. However, Fig 5D shows an RMP of -85 mV. This suggests that, if corrected, it would be around -100 mV.

4. The discussion regarding contribution of LTCS to memory is highly speculative and does not take into account network activity. It is recommended that the discussion be revised to limit highly speculative comments.

5. Results section on page 12 of PDF: Please clarify the cell numbers relevant to the statement "In the cells that display LTCS, the decay time course in WT GCs was slightly lower than the decay time course in cav3.2KO mice (WT: 83.98+/-15.77ms n=13)". Shouldn't the N=16? Earlier in this section, it was reported that LTCS was found in 16/20 immature granule cells (i.e., 80% as per Figure 2B).

6. Figure 1: Consider adding a dashed border between the outer granule cell layer and inner molecular layer to help visualize dendrites in the molecular layer. Also include color-coded text beside or superimposed on the top photomicrograph to indicate each stain (e.g., Neurobiotin-488 in green font, DCX in pink font, and NeuN in blue font) and illustrate lack of DCX labeling in mature DGGC. Additionally, example of outward rectifying cells needs to be included.

7. Figure 5 and 6: Include average firing rates. Also, as with Figure 4, include captions indicating "Intermediate" and "Mature" in the title of the figures 5 and 6.

8. Results section pertaining to Figure 3C (page 13 of PDF): Please include "mature" after N=14 when discussing results for Ro.

9. Please review the paper for typos (Figure 8D &I: "Resonance Strenght") and capitalization of companies or products (e.g., Alomone)

10. Results section related to Figure 7 (page 18 of PDF file): Please indicate "7Ei" and "7Eii" when reporting graphs instead of simply 7E.
