# Supplementary figures and images for: Maturational Stage-Dependent Contributions of the Cav3.2 T-Type Calcium Channel to Dentate Gyrus Granule Cell Excitability
Source: eNeuro. 2025 Apr 1;12(4):ENEURO.0423-24.2025. doi: 10.1523/ENEURO.0423-24.2025 (PMC11974363; doi:10.1523/ENEURO.0423-24.2025)

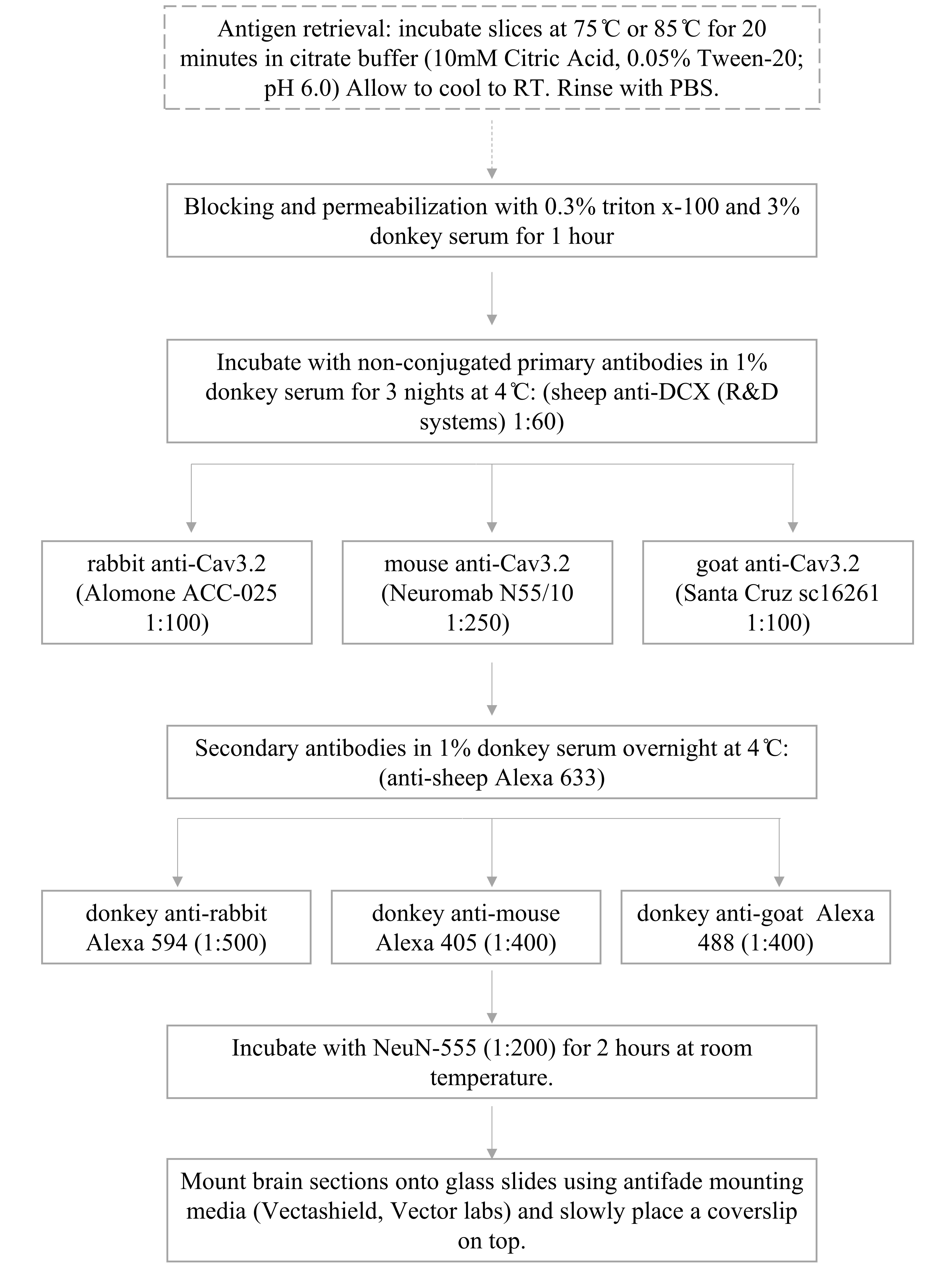

Supplement: Figure 1-1 — Flowchart of immunohistochemistry protocols used to localize Cav3.2 channels in the DG. Following transcardial perfusion with paraformaldehyde (PFA), brains were dissected, placed in 4% PFA overnight at 4 °C and then sucrose (10% overnight, followed by 30% overnight) for cryoprotection. Fixed brains were subsequently embedded in optimal cutting temperature (O.C.T) compound and stored at -80 °C. Tissue was sliced into 40 µm thick coronal sections or magic cut sections using a cryostat. Sections were washed in PBS and stored in storage solution (0.81 M sucrose, 30% ethylene glycol in 0.1 M PBS) at -80 °C until use (Potts et al., 2020). Free floating sections were washed with PBS prior to either antigen retrieval or directly to permeabilization and blocking (RT: room temperature). Download Figure 1-1, TIF file. [file eneuro-12-ENEURO.0423-24.2025-s002.tif]

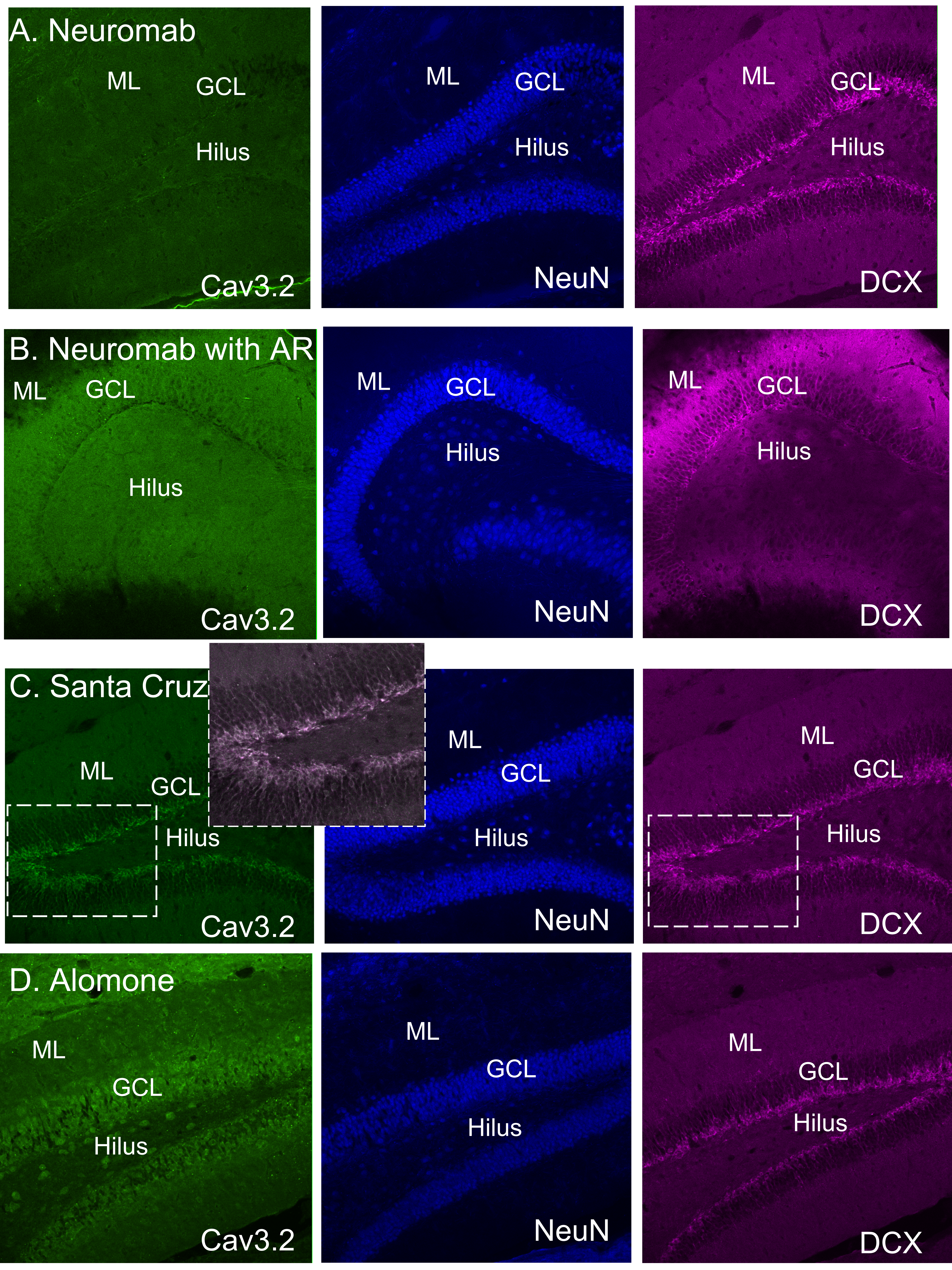

Supplement: Figure 1-2 — Immunohistochemical staining in the DG, comparing different antibodies targeting Cav3.2 channels. Three different commercial antibodies recognizing distinct epitopes were systematically tested in both WT and KO mice brain slices. The Neuromab antibody (A) did not show any immunoreactivity for Cav3.2 channels in the DG or other brain regions examined (not shown). Antigen retrieval (AR) method (B) resulted in non-specific background fluorescence with no improvement of Cav3.2 immunolabeling with Neuromab antibody. Although immunofluorescence was observed in GCs using the Santa Cruz antibody (C), the staining matched and overlapped entirely with that obtained using doublecortin (DCX), indicating cross-reactivity (insert shows superimposed Cav3.2 DCX staining). Lastly, by using the Alomone antibody (D), non-specific immunolabeling was detected in hilar cells and some inner GCs. All antibodies showed a similar pattern in slices from KO mice (not shown). We further observed diffuse, non-specific staining in other regions of the brain known to express Cav3.2 channels (not shown) (ML = molecular layer. GCL = granule cell layer). Download Figure 1-2, TIF file. [file eneuro-12-ENEURO.0423-24.2025-s003.tif]
